# An experimental, behavioral, and chemical analysis of food limitations in mutualistic *Crematogaster* ant symbionts inhabiting *Macaranga* host plants

**DOI:** 10.1002/ece3.9760

**Published:** 2023-02-07

**Authors:** Mickal Y. I. Houadria, Giulio Barone, Tom M. Fayle, Thomas Schmitt, Petr Konik, Heike Feldhaar

**Affiliations:** ^1^ Biology Centre of the Czech Academy of Sciences Institute of Entomology Ceske Budejovice Czech Republic; ^2^ Department of Agricultural, Food and Forest Sciences University of Palermo Palermo Italy; ^3^ School of Biological and Behavioural Sciences Queen Mary University of London London UK; ^4^ Department of Animal Ecology and Tropical Biology, Biocentre University of Würzburg Würzburg Germany; ^5^ Department of Chemistry, Faculty of Science University of South Bohemia in Ceske Budejovice Ceske Budejovice Czech Republic; ^6^ Animal Population Ecology, Animal Ecology I, Bayreuth Center of Ecology and Environmental Research (BayCEER) University of Bayreuth Bayreuth Germany

**Keywords:** behavior, coccids, food bodies, GC–MS, isotope, myrmecophyte, plant–herbivore interactions, proteomics

## Abstract

Obligate mutualistic plant–ants are often constrained by their plant partner's capacity to provide resources. However, despite this limitation, some ant partners actively reject potential prey items and instead drop them from the plant rather than consuming them, leaving the ants entirely reliant on host plant‐provided food, including that provided indirectly by the symbiotic scale insects that ants tend inside the plants. This dependency potentially increases the efficiency of these ants in defending their host. We hypothesize that if this ant behavior was beneficial to the symbiosis, prey rejection by ants would be observed across multiple plant host species. We also hypothesize that plant‐provided food items and symbiotic scale insects from other ant plants should be rejected. We address these hypotheses in the *Crematogaster* ant–*Macaranga* plant system, in which plants provide living space and food, while ants protect plants from herbivory. We observed food acceptance and rejection behavior across five ant species and three plant host species. Ants were offered three types of food: termites as a surrogate herbivore, symbiotic scale insects, and nutritious food bodies (FB) produced by different host plant species. The unique ant species living in *M. winkleri* was the most likely to reject food items not provided by the plant species, followed by ants in *M. glandibracteolata*, while ants in *M. pearsonii* accepted most items offered to them. Using stable isotopes, chemical cues, and proteomic analyses, we demonstrate that this behavior was not related to differences between plant species in nutritional quality or composition of FB. Isotopic signatures revealed that certain species are primary consumers but other ant species can be secondary consumers even where surrogate herbivores are rejected, although these values varied depending on the ant developmental stage and plant species. *Macaranga pearsonii* and *M. glandibracteolata*, the two most closely related plant species, had most similar surface chemical cues of FB. However, *M. glandibracteolata* had strongest differences in food body nutritional content, isotopic signatures, and protein composition from either of the other two plant species studied. Taken together we believe our results point toward potential host coercion of symbiont ants by plants in the genus *Macaranga* Thouars (Euphorbiaceae).

## Graphical abstract

Understanding why within an ant–plant system, some symbiotic ants have different behaviors and diets. We demonstrate that this depends on the plant host rather than the ant species.

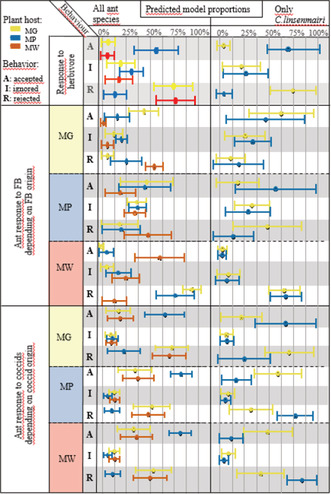

## 1 INTRODUCTION

All organisms on earth are embedded in complex networks of interactions. Mutualisms involve evolutionary adaptations, in which partners invest in costly traits that benefit their partners (Ferriere et al., 2002). These positive interactions underlie a wide range of ecosystem functions (Frederickson, 2017). Nutrition is often traded in the widespread and ecologically important symbiotic mutualistic interactions between ants and plants. These relationships can range from obligate to facultative interactions. Ant–plants provide ants with food in the form of food bodies (FB) and/or extrafloral nectaries (EFN) and with shelter, in form of domatia (Fiala & Maschwitz, 1992; Heil, Hilpert, et al., 2001; Itino et al., 2001; Sagers et al., 2000). Plant‐provided living space (domatia) consists mostly of hollow structures that are generated from the stem, leaves, or spines/thorns (Nelsen et al., 2018). In return, ants defend host plants from herbivores and in certain cases also clean plants of encroaching vines and fungal spores (Eck et al., 2001; Federle et al., 2002; Maschwitz et al., 1989). However, all mutualisms are vulnerable to cheating, in which one partner does not provide benefits, but continues to receive them (Ferriere et al., 2002).

This has two important evolutionary ecological implications. First, plants should direct food (FB, EFN) to the most beneficial ant partner (Giron et al., 2018), hence avoiding ants that exploit resources without reciprocating (Heil et al., 2009). This can be achieved through the growth of physical barriers that only more beneficial ant partners are able to pass (Federle et al., 1997), or even chemical barriers in the form of protease inhibitors rendering plant‐provided food digestible only for beneficial partners (Orona‐Tamayo et al., 2013). Second, in addition to preventing the “wrong” partner from exploiting plant‐provided food, it is necessary to ensure that the “right” partner continues to provide protection services. One strategy that plant hosts use to ensure that they receive protection benefits in return for their nutritional investment in their ant symbionts is sanctions, for example, where plants reduce food provisioning in response to increased herbivore damage (Orona‐Tamayo & Heil, 2013). However, such mechanisms are only likely to evolve when the food provided by the plant is indispensable for the ants because otherwise the ant partners could gather food elsewhere. In certain cases, it has been shown that plants use coercion/manipulation of the ant partner to prevent feeding on other food sources (Nepi et al., 2018). For example, in the *Acacia–Pseudomyrmex* interaction, ant workers cannot digest any source of sugar other than the one provided by the plant through EFN. This is because EFN sugar contains specific inhibitors of digestive enzymes that are needed for digesting other food sources, effectively “addicting” the ant partner to that food source (Heil et al., 2014). Hence, despite abundant and accessible alternative food sources (e.g., EFN from another plant and honeydew from Homoptera), which are potentially more valuable in terms of nutrients, the ants do not use such food sources. Interestingly, in many other ant–plant systems, ants tend symbiotic scale insects (Homoptera: Coccidae) that feed on plant phloem sap while living inside the domatia. In this type of tripartite mutualism, coccids provide carbohydrate‐rich honeydew (at the plant's expense) while ants protect coccids from predators. However, it is not clear whether the coccids that live symbiotically with the ants are themselves used as food. Interestingly, in the few studies on dietary preferences of symbiotic ants tending coccids (Fiala & Maschwitz, 1990; Heckroth et al., 2001), ants did not consume them and only tended them for honeydew. In certain ant–plant systems (*Cladomyrma* ants: Maschwitz et al., 1989), honeydew and EFN may be the only source of food for the ants, presenting challenges for the development of the larvae on such protein‐deprived diets (Ribeiro et al., 2019). Many arboreal ants have highly specialized digestive systems characteristic of primary consumers due to the need to acquire sufficient nitrogen from carbohydrate‐rich diets (Davidson et al., 2003; Feldhaar et al., 2007; Mayer et al., 2014). However, such high specialization could come at the cost of limiting diet breadth. This could explain why some obligate symbiotic ants drop herbivores off the plant, rather than consume them (Moog et al., 2005). Moreover, FB and EFN are presented on parts of the plant vulnerable to herbivory such as new stems and young leaves. Compelling ants to patrol the plants in these specific locations may considerably improve the services provided by the ant to the plants (Moog, 2009).

In our tripartite *Macaranga* plant*—Crematogaster* ant*–*coccid study system—, the ants also tend specialized coccids. However, in contrast to *Cladomyrma*, where plants only provide EFN, and where prey rejection has been observed*, Macaranga* plants also provide protein‐ and lipid‐rich FB to their ant partners (Ueda et al., 2010). A rejection of potential insect prey items has also been observed in the *Macaranga–Crematogaster* system, as well as a rejection of plant‐based FB collected from other plant genera (Fiala & Maschwitz, 1990). *Macaranga* species show a range of interactions with ants from facultative to obligate. Among the latter, most symbiotic ants are of a closely related group of *Crematogaster* (*Decacrema*) ants (Feldhaar et al., 2016), each of which can colonize several different *Macaranga* species. These interactions can also be exclusive, with an ant species only being found on one plant host species and vice versa. Previous research has already shown the importance of food quality in relation to obligate and facultative interactions, where plants involved in facultative mutualisms provide lower‐quality food (Heil, Fiala, et al., 2001). We take this a step further and analyze obligate systems in relation to the degree of specialization (host specificity) and food limitation of the ants. The only previous work on the food limitations of symbiotic *Macaranga* ants was based on a single ant–plant system with a small number of colonies maintained in captivity (Fiala & Maschwitz, 1990). This work was an exploration of what these ants could be fed to survive in captivity, rather than to investigate why these ants have such feeding behaviors. Hence, the drivers of food limitations for ants in this system remain unknown. Here, we analyze how feeding behavior of *Crematogaster* ants differs across different combinations of ant and *Macaranga* species.

We hypothesize that plant ants limit the use of non‐host food sources, either (i) because the ants prefer the high‐quality food provided by the plant host regardless of their specialization level, or (ii) due to a high level of dietary specialization (that may be due to host coercion) limiting their capacity to consume sources of food other than honeydew and plant‐provided food from the specific host they have colonized.

To test these hypotheses, first, we explore whether food quality drives feeding behavior in these obligate plant ants. To characterize the quality of FB provided by the plants, we measured nitrogen content and FB‐specific protein composition (Heil et al., 1998; Orona‐Tamayo et al., 2013). We predict that herbivore rejection rate should be higher on plants with FB of higher quality as ants should be less limited in nitrogen on such plants. In addition, these ants would derive a higher proportion of nitrogen directly from the plant instead of prey items and should thus have a relatively lower trophic enrichment than ants accepting them. We, therefore, characterized stable isotope signatures (δ15N and δ13C) of FB, ants, and symbiotic scale insects as potential herbivores that may be consumed by the ants. Ants should show similar behaviors in terms of acceptance or rejection of experimentally provided FB when FB have similar FB protein composition.

Second, we analyzed whether feeding behavior is driven by the plant partner. If this was the case, we would expect ants to show a higher rejection rate of food sources not provided by the specific host, either due to coercion by the plant host or dietary specialization by the ants. Partner recognition (Grasso et al., 2015) should be most developed in the most exclusive interactions (one plant species and one ant partner), and therefore, those ants should display the strongest rejection behavior toward any food not provided by their plant host. These patterns should be observed irrespective of the quality of the food. As rejection/acceptance mechanisms of plant‐provided food may be mediated through chemical surface cues (Jürgens et al., 2006; Souto‐Vilarós et al., 2018), or specific protein composition of FB, the reaction of symbiotic ants toward more similar plant‐derived food sources (in terms of chemical composition) should be more alike.

## 2 MATERIALS AND METHODS

### 2.1 Study sites

Fieldwork was conducted from February to March 2018 along logging roads in lowland rain forest in Sabah, Malaysian Borneo. The area had been selectively logged twice between 1980 and 2000, and then heavily logged in 2013–2015. This area is part of the Stability of Altered Forest Ecosystems project (SAFE project habitat “matrix”; Ewers et al., 2011). The climate in Sabah is relatively unseasonal, with mean annual precipitation of 2880 mm and 80%–90% relative humidity. Daily temperatures range from 19 to 34°C (annual mean: 26.9°C). Rainfall tends to be highest from November to February.

### 2.2 Choice of *macaranga* species and species identification

We selected three different species of *Macaranga* frequently found in heavily logged forest. *Macaranga pearsonii* (MP) and *Macaranga glandibracteolata* (MG) are two phylogenetically closer species (sections *Pruinosae* and *Pachystemon*, respectively: Bänfer et al., 2004; Davies, 2001), each colonized by multiple (two to four) closely related symbiotic ant species belonging to the *Crematogaster* subgenus *Decacrema* (Feldhaar et al., 2016). Since these two *Macaranga* species share ant symbiont species, *C. linsenmairi* (Table 1), we were able to compare behavior of the same ant species between different plant species. This was the only ant species with enough replication to do this. *Macaranga winkleri* (MW) belongs to a more distantly related group (section *Winklerianae*; van Welzen et al., 2014) and is colonized by a single completely specific ant species, *Crematogaster* sp. 8, that belongs to a different subgenus from the other symbiotic ant species (Fiala et al., 1999).

**TABLE 1:** Summary of sample sizes for all analyses, including the numbers of colonies of the different ant species found in the different tree species and different sample sizes depending on the species or food item type.

Sample type	Behavior	GC–MS	Isotope	Proteomics
Termites	28			
	32			
	29			
*C. linsemairi*	5			
	8			
*C. borneensis*	3			
	2			
*C. captiosa*	1			
*C. maryatii*	1			
*C. sp. 8*	10			
Workers		10	10	
		10	9	
		11	9	
Larvae			10	
			9	
			7	
Coccids MW	38		6	
	22			
	33			
Coccids MP	41		10	
	22			
	36			
Cocc. MG	8		6	
	19			
	20			
FB. MP	31	10	10	12
	26			
	32			
FB. MG	28	10	9	12
	27			
	28			
FB. MW	32	10	9	12
	29			
	29			

*Note*: Blue, gray, and red colored squares represent, respectively, the different Macaranga species MP, MG, and MW.

Trees selected were 2–4 m in height with no apparent significant damage either from vertebrates (elephants, human‐cut trails) or from invertebrate herbivores, and all displayed high levels of ant activity, which was defined as at least five ants per minute circulating from the apex of the branch to a domatium (see Table 1 for plant/ant species associations and sample sizes, which vary between different analyses). The three plant species were identified following Davies (2001). For ant identification, up to three workers (and a queen when collected) of each morphotype from each tree were point mounted, photographed, relevant traits measured (Dino‐Lite 2.0), and were identified to species level using the latest systematic key on *Macaranga‐*associated *Crematogaster* ants (Feldhaar et al., 2016). The single easily recognizable ant species in MW (*Crematogaster* sp. 8) was identified in the field.

### 2.3 Food items

Surrogate herbivores, scale insects, and FB were offered as food items. We selected termites as surrogate herbivores rather than true *Macaranga* herbivores because of their low mobility and easy access to colonies of the genus *Dicuspiditermes* enabling standardization. We used only mature workers (not nymphs) and did not use soldiers since these have defensive mechanisms that might affect ant behavior. Symbiotic scale insects from the genus *Coccus* and FB were extracted from the *Macaranga* species we specifically studied but on which no behavioral experiments were conducted since extraction involved partial destruction of plants. Ants may respond differently depending on the symbiotic coccid species (Heckroth et al., 1998, 2001). Identification of coccids in the field was not possible, but to account for this we sampled and offered coccids from all three different plant species in each behavioral experiment. Scale insects were carefully removed from inside domatia using soft forceps in order to prevent damage, and were kept in 5‐ml Eppendorf tubes. Only visibly intact scale insects that had reached the third or later instar were used as food items. FB were extracted from stipules of the different plant species with soft forceps and were kept in 5‐ml Eppendorf tubes in a cool box at 10°C until experimental placement.

### 2.4 Behavioral responses to different food items

Food items were placed at ~10 cm intervals on the accessible branch where ant patrolling was most frequent. All three different food items were displayed simultaneously in random order. A single branch was used per tree. Live food items (termites and coccids) were simply deposited and because of their low mobility they rarely fell off. When the branch angle was too steep to rest food items on (*n* = 3 trees, each of a different species), we bent the tree using a rope (maximum of 45°) 24 h prior to the experiment, thus minimizing disturbance due to shaking of the branch. If an item was not encountered after 7 min, then it was moved to a new location on the same branch. After a food item was encountered (through antennation), ant behavior was noted (see below), then a new food item was placed a few centimeters from the previous one until three replicates of each food item were obtained for each tree. Coccids from each plant species were placed four times to increase replication to account for potential variability in response to different species and instars. We considered an item to be *accepted* when the ants carried it inside the hollow stem (domatia) of the tree, or if it was consumed where it was found. An item was recorded as *rejected* when it was thrown off the tree. *N/A* was recorded if an ant simply carried the item but was not observed to throw it off, consume it, or bring it inside the tree. If the food item was encountered (through antennation) but not picked up, it was considered *ignored*. Experiments were conducted from 8:00 a.m. to 12:00 p.m. in the absence of wind or rain and when the plant's surface was dry.

### 2.5 Chemical analyses

For all chemical analyses, ant workers were sampled directly from the surface of plant branches and FB were collected from the stipules closest to the plant's apex. To retrieve scale insects (tightly attached with proboscis) as well as ant larvae within the hollow stem (domatia), we partially dissected trees after the behavioral experiments were conducted.

#### 2.5.1 C13 and N15 isotopes and C/N ratios

We measured stable isotopes δC13 and δN15 to characterize the trophic level of the different ant species' developmental stages (worker/larvae) in relation to host plant FB and coccids (primary consumers and potential ant prey). For each category (ants, larvae, coccids, and FB), a minimum of five items/individuals from each plant host were placed in Eppendorf tubes with cotton wool and silica gel in sufficient quantity to completely dehydrate the sample. Measurements of δC13 of ants that were raised entirely on a synthetic diet with known isotope ratios (Feldhaar et al., 2010) predict that ants should be enriched by about 1% to 1.2% in comparison to their diet. The most likely source of C would be nectar from EFN or honeydew produced by the coccids. However, since we were unable to collect these two potential food sources we were unable to measure the contribution of these sources to the δ13C signature of the ants.

For our study, we believe that δ15N is more interesting since we need this value to interpret the patterns observed in rejection of food items that should be collected to enhance intake of protein. Earlier studies have shown that ants on *Macaranga* are limited more by N than by C as fertilizing *Macaranga* plants results in increased food body production (with high N‐content) and subsequently enhanced ant colony growth (Heil, Fiala, et al., 2001). We, therefore, consider the C/N ratio a good measurement of plant investment in food body quality.

Before stable isotope analysis, all samples were oven‐dried overnight at 105°C. Relative C and N isotope natural abundances were measured in dual‐element analysis mode with an elemental analyzer (1108; Carlo Erba Instruments) coupled to a continuous‐flow isotope ratio mass spectrometer (delta S, Finnigan MAT) via ConFlo III open‐split interface (Thermo Fisher Scientific) as described in Bidartondo et al. (2004). For further details, please refer to Appendix S1 *Proteomics*.

We hypothesized that variability in ant food preference is based on the protein composition of the FB, with FB with more similar composition being treated by the ants in a similar way. FB may contain enzymes that strongly influence their digestibility in the ants. Either a protease inhibitor would block ant digestive enzymes making it unable to digest other protein sources (Heil et al., 2014) or the FB has inhibitors preventing digestion by non‐mutualistic species (Orona‐Tamayo et al., 2013). In both cases, this inhibitor would have to be fast acting and readily available during the first stages of the digestion process. For this reason, we digested the FB stepwise, and we expected the inhibitor to be present in the 30 min digest, and depleted or missing in the 12‐, 24‐, and 36‐h digestions. We extracted a minimum of 15 FB from 12 trees for each plant species. After collection from the plant, FB were directly placed in PTFE vials and installed in a dry shipper at −50°C (CHART Biomedical MVE SC 4/2) until brought back to the laboratory. The LC/MS analysis was performed on a NanoAcquity UPLC coupled online to the ESI Q‐Tof Premier mass spectrometer (both Waters). Protein composition was based on retention time of peptide migration through column based on peptide hydrophobicity. A minimum of two peptides matched to a protein was considered a positive result; four digestion times (30 min, 12, 24, and 36 h) were performed with fresh trypsin, and each sample was measured six times. For further details on protocol and protein identification, see Appendix S3 and Tables S1 and S2.

#### 2.5.2 Chemical cues

To compare acceptance of FB by ants in relation to differences in FB chemical profiles, external food body chemicals were extracted and chemical profiles were compared across host plant species. Chemical cues from ants were also extracted to describe any potential contamination of the chemicals present on the FB. We extracted a minimum of 10 FB and five ants per sample from 10 trees for each plant species. Samples for chemical cues were placed in 1.5‐ml glass vials with 1.5 ml of hexane for 10 min. The hexane was then transferred to another 1.5‐ml glass vial. Extracts were shipped to the University of Würzburg and were analyzed with an Agilent 6890 gas chromatograph coupled with an Agilent 5975 Mass Selective Detector (Agilent). For further details, see Appendix S2.

### 2.6 Data analysis

All R analyses were conducted on R version 1.2.5033.

#### 2.6.1 Behavioral responses to different food items

As detailed at the end of the introduction, we first explored the rejection of food items by ants depending on the plant host by using regression models with ordered categorical response variables. Behavioral responses were ordered by level of attraction to the food item. The order was as follows: R < I < A, where “R” denotes rejection of the food item which meant it was thrown off the tree, “I” denotes ignored, and “A” accepted. As reactions of ants toward different food types showed high levels of differences regardless of the plant, we performed separate analyses for each type of food. For the response to surrogate herbivores (termites), explanatory variables were the plant host species and tree identity as a random factor. Each tree is usually colonized by a single ant colony and we, therefore, account for colony‐specific behavior by including this random effect. For both FB and coccids, the explanatory variables were the same as those for the surrogate herbivore analyses, but with the addition of interactions between both the plant species of origin of FB/coccids (which plant species they were collected from) and the plant host species (where the ants were living). Replication was at the level of 10 trees per plant species and at least three identical food items per tree, and trees as a random factor was included to account for the non‐independence of trials within a tree. We could not include ant species identity in these models since MW has no symbiotic ant species in common with the other two plant species. However, we did rerun all models separately for the ant species *C. linsenmairi*, which was common to both MP and MG (five and eight plants, respectively, Table 1). We used R package “*tidyverse*” (Wickham et al., 2019) for data management and plotting (ggplot() and ddply()). For modeling, we then used clmm() from the “*ordinal*” package (Christensen, 2015) and ggpredict() from the “*ggeffects*” package (Lüdecke, 2018) for data visualization.

#### 2.6.2 Isotopic analyses

We hypothesized that acceptance of plant‐produced FB may be driven by their quality. We, therefore, first characterized food body quality based on the amount of nitrogen in comparison to carbon content (C/N ratio). As ants are limited in nitrogen, FB with lower C/N ratio should be preferred and, in turn, ants should reject most other food items presented to them which would result in a higher rejection rate of surrogate herbivores as a potential source of nitrogen. We expect that ants reject surrogate herbivores to have a relatively lower trophic enrichment (δ15N) than those which accept them, i.e. they feed on FB directly provided by the plant to a larger extent. This effect should be most pronounced in the larvae, which need higher protein intake. We, therefore, conducted additional analyses to explore whether trophic enrichment of the different ant species varied between tree hosts and across ant development stages (larvae and worker) accounting for variance in potential food resources (coccids and FB from each plant host were considered as baselines); we collected a minimum of nine and six replicates for coccids and FB, respectively (Table 1). For each analysis, we had at least nine replicates (different colonies in different trees) except for the larvae of MW where we had only seven replicates of larvae (Table 2). The proportion of different food sources consumed by the ants or larvae was calculated by solving the likelihood equations for mixtures of distributions for stable isotopic data within a Bayesian framework, using the “*simmr*” R package (simmr_load(), simmr_mcmc(), and compare_sources()). For more information on the Bayesian mixing model, please refer to Parnell et al. (2013). The two potential ant food sources (FB and coccids) were considered to reflect primary producers (since FB are produced by the plant) and primary consumers respectively (coccids as herbivores). These analyses focused mainly on differences in N isotopes reflecting different sources of amino acids or protein (a more limiting factor than carbohydrates; Heil, Fiala, et al., 2001). We, therefore, did not consider honeydew as a source because of its very low nitrogen concentrations (Ribeiro et al., 2019) and sampling limitations. We conducted in R pairwise *t*‐test comparisons of isotopic ratios within a species and *t*‐test comparisons between species (Table 2).

**TABLE 2:** Comparison of δ15N among FB, ants, ant larvae, and coccids within a plant host species (pink) using pairwise *t*‐tests, and for the same item type across plant host species (gray) using *t*‐tests (^n.s^ *p* > .05; *p < .05; **p < .01; ***p < .001).

	δ15N	MP				MG				MW		
		FB	Ants	Larvae	Coccids	FB	Ants	Larvae	Coccids	FB	Ants	Larvae
MP	Ants	n.s										
	Larvae	*	*									
	Coccids	n.s	*	***								
MG	FB	*										
	Ants		*			n.s						
	Larvae			*		**	**					
	Coccids				n.s	n.s	**	***				
MW	FB	***				**						
	Ants		**				***			*		
	Larvae			*				**		*	n.s	
	Coccids				n.s				n.s	*	n.s	n.s

#### 2.6.3 Proteomics analyses

In order to explore if plants with similar FB protein composition show similar ant behaviors, a two‐way crossed PERMANOVA analysis was performed with *Macaranga* species and digestion time in the proteomic analysis as fixed effects and composition of peptides (lengths and quantity) obtained by enzymatic digestions of the FB as the response variable. One‐way PERMANOVA analyses were also conducted within each digestion time. We had six replicates per digestion time and plant species. We visualized these results using NDMS plots. PERMANOVA and NMDS analyses were performed using Primer V.6 (Clarke & Gorley, 2006).

#### 2.6.4 External chemical cues of FB

To analyze if symbiotic ants on closely related *Macaranga* species respond more similarly to food items with more similar surface chemical cues, we compared the profiles of FB using a one‐way PERMANOVA design (Primer V.6) where we used the chemical cue results as response variable and *Macaranga* species as a fixed factor and tree individual as a random factor. We visualized these results using NDMS plots (Primer V.6). We extracted FB from a minimum of 10 plants from each species.

## 3 RESULTS

### 3.1 Behavioral response to different food items

#### 3.1.1 Termites as surrogate herbivores

**FIGURE 1:**
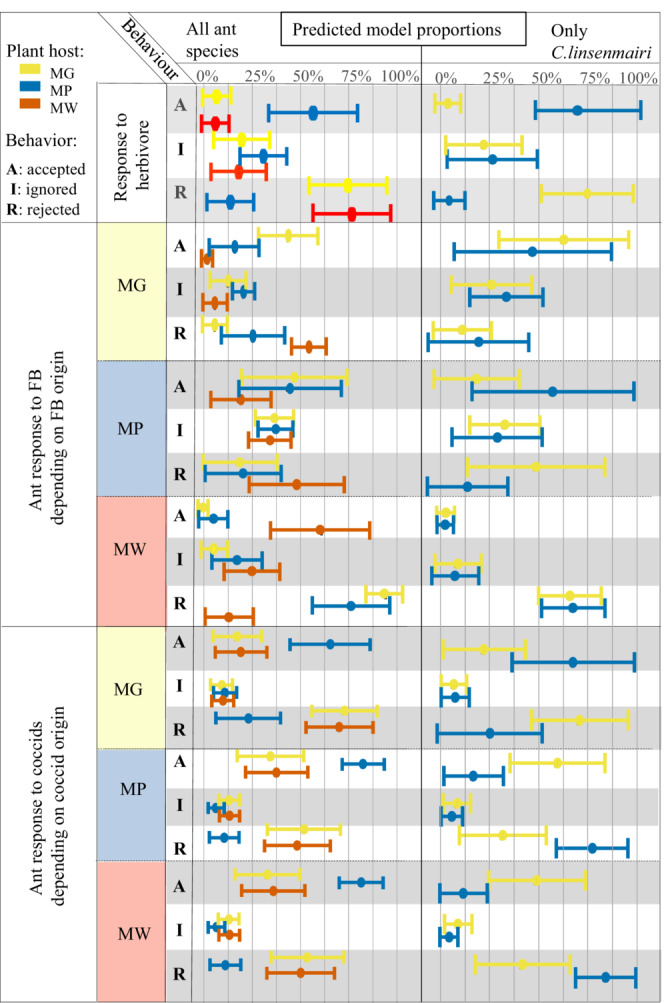
Behavioral responses of symbiotic ants to different food items experimentally placed on *Macaranga* trees (A = Accepted; I = Ignored; R = Rejected) across food types (termites, FB, and coccids) and plant species of origin for the food. Results are presented for all ant species combined, and separately for *C. linsenmairi*, the only ant species found in sufficiently many trees for comparisons to be made between species (tree species MG and MP). See Tables 2 and 3 for statistical tests.

Ants living in *M. glandibracteolata* (MG) and *M. winkleri* (MW) hosts rarely accepted the insect herbivore (<10% of occasions), whereas ants in *M. pearsonii* (MP) trees accepted them significantly more often (>50% of occasions; Figure 1; Table 3). There were no significant differences in the proportion of surrogate herbivores ignored on different *Macaranga* species.

**TABLE 3:** Comparison of behavior (ordered categorical response variable) of ant symbionts when presented with termites (surrogate herbivores) and coccids in relation to plant host species and for coccids, the host plant of origin.

	Host plant comparison	*z* value	*p*	Comparison of origin	*z* value	*p*
Coccids	MP‐MW	4.5	***	MP‐MW	0.21	n.s
	MG‐MW	0.3	n.s	MG‐MW	4.91	*
	MP‐MG	4.54	***	MP‐MG	2.28	*
	*L*: *MP‐MG*	2.8	**	*L: MP‐MG*	1.13	n.s
				*L: MP‐MW*	2.77	n.s
				*L: MG‐MW*	0.71	n.s
Termites	MP‐MW	3.7	***			
	MP‐MG	3.6	***			
	MG‐MW	0.15	n.s			
	*L: MP‐MG*	3.47	***			

*Note*: Plant individual was included as a random variable. Significance values are denoted as follows: ^n.s^ *p* > .05; *p < .05; **p < .01; ***p < .001. *L* and italic font indicate comparisons conducted only on *C. linsenmairi*. See Figure 1 for visualization of results.

#### 3.1.2 Food bodies

Ants living in MG and MW tended to accept homospecific FB (over 40% and 50%, respectively) and rejected or ignored heterospecific FB (up to 90%). However, ants on MP accepted roughly similar proportions (~45%) of FB from MP and MG (Figure 1; Table 4).

**TABLE 4:** Comparison of behavior (ordered categorical response variable) of ant symbionts when presented with FB as predicted by plant host species, plant species of FB origin, and their interaction (fixed effects).

Host plant comparison	FB comparison origin	*z*‐value	*p*
MP‐MG	MP‐MG	2.13	*
	MP‐MW	1.253	n.s
	MW‐MG	3.12	**
	*L: MG‐MW*	0.38	n.s
	*L: MP‐MW*	1.65	n.s
	*L: MP‐MG*	2.27	*
MG‐MW	MP‐MW	5.64	***
	MP‐MG	3.38	***
	MW‐MG	7.38	***
MP‐MW	MP‐MW	5.03	***
	MP‐MG	1.63	n.s
	MW‐MG	5.74	***

*Note*: Plant individual is included as a random effect. Significance values are denoted as follows: ^n.s^ *p* > .05; *p < .05; **p < .01; ***p < .001. *L* and italic font indicate comparisons conducted only on *C. linsenmairi*. See Figure 1 for visualization of results.

#### 3.1.3 Coccids

Coccids from MG plants were the only ones for which ant responses differed between plant hosts (Table 4). MP ants, in general, accepted coccids (~75%) more than ants on the other plants, and rejection rates were very similar for MG and MW ants (Figure 1; Table 3) regardless of coccid origin (from 50% up to 65% for coccids originating from MG). When comparing the same species (*C. linsenmairi*) found in both MP and MG, there was a reverse effect (Figure 1; Table 3) with MP ants accepting more coccids originating from MG plants, but MG ants accepting more coccids from both MP and MW (50% and 60%).

### 3.2 C/N ratio and isotopic signatures

#### 3.2.1 C/N ratio

**FIGURE 2:**
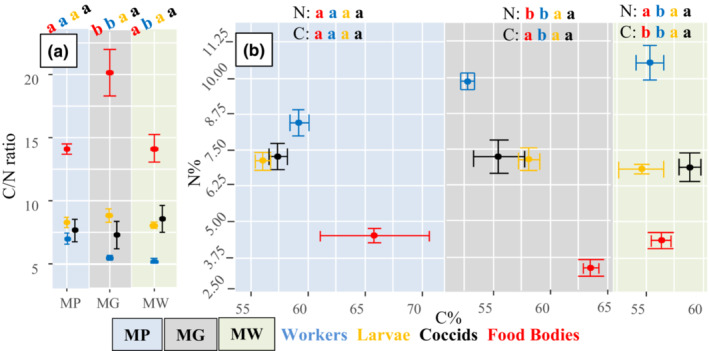
Differences in C and N composition of ant workers, ant larvae, coccids, and FB across the three *Macaranga* species studied. (a) Differences in C/N ratio, calculated as carbon content divided by Nitrogen content across plant species. High C/N ratios indicate low nitrogen content. (b) Differences in N and C content across plant species. Letters at the top of each panel represent comparisons (*t*‐tests) of the same item in different plants, with significantly different results being denoted by differing letters.

In general, workers had a lower C/N ratio (higher N concentration) than larvae. Ant larvae across the different plant species had similar ratios, but workers from MP had higher C/N ratios than ants from the two other plant species (Figure 2a). The C/N ratio of FB was much higher than that of workers and larvae. FB from MG had the highest C/N ratio, with those from MP and MW being similar and lower.

#### 3.2.2 N and C content

MG FB contained the lowest percentages of N (Figure 2b). Larvae and coccids did not differ in N or C percentage depending on the plant host. However, MP worker ants had lower N percentages than worker ants from the other two plant species and MG had lower C percentages than ant workers from the other two species. FB from MW had the lowest C percentages compared to the other two plant species.

#### 3.2.3 δΝ15

Only on MW did worker ants have higher *δΝ15* than their FB, but all larvae showed higher *δΝ15* than their FB regardless of the host (Table 2). *δΝ15* levels of larvae and ants were higher than those of their coccids for both MG and MP but not in MW (Table 2).

#### 3.2.4 δC13

**FIGURE 3:**
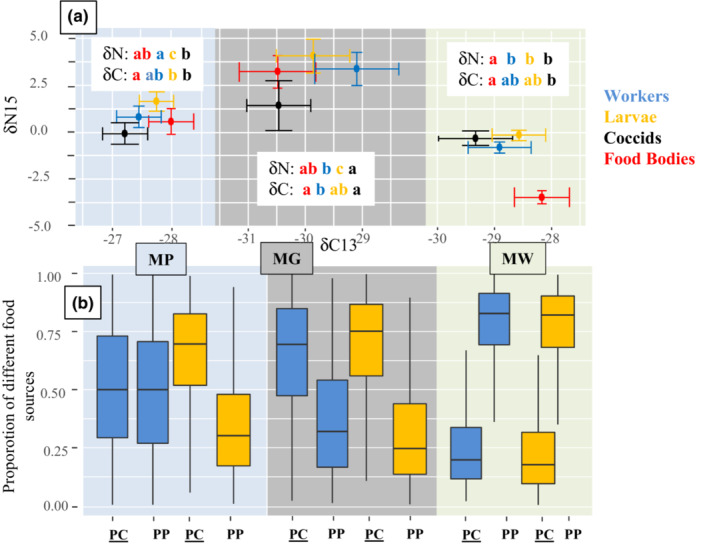
(a) Trophic levels inferred from δ15N and δ13C ratios for ant workers, ant larvae, symbiotic coccids, and plant‐provided FB across the three different plant species. Significantly different isotope ratios (as assessed by pairwise *t*‐tests) are denoted by differing letters. For comparisons of δ15N between different items within and between plant species, see Table 2. (b) Results of stable isotope mixing models considering primary consumers (PC) and primary producers (PP) as food sources for both larvae and workers. 0 indicates no contribution of that specific food source while 1 indicates that the food source comprises 100% of the diet.

This did not differ depending on the plant host for larvae or ant workers (Figure 3a).

#### 3.2.5 Diet contribution

Based on stable isotope signatures (*δ13C* and *δ15N*), MW larvae and ants have a diet primarily based on the FB provided by their host plant (Figure 3b), whereas for ants and larvae in MG, coccids make a greater contribution as a source of food (more pronounced in ant larvae). Similar *δ15N* enrichment as in MG larvae was observed in MP larvae, although the worker ants in MP had stable isotope signatures pointing toward equal contribution of the two food sources.

### 3.3 Protein composition

The two‐way crossed PERMANOVA analysis showed protein composition differences between FB of different species, digestion time, and the interaction between these two factors. The 30 min digestion showed most pronounced differences (PERMANOVA; Table S1; Figure S2A). Within this digestion time, all species had different protein profiles (Figure S2B, PERMANOVA: *df* = 2; pseudo‐*F* = 19.68; *p* < .001), suggesting differences in the digestion of different FB. Interestingly, protein composition of FB from MW and MP was more similar, despite MP and MG being taxonomically closer (Table S1).

### 3.4 Chemical cues

The chemical profiles of the FB from different plant species were all significantly different (PERMANOVA: *df* = 2; pseudo‐*F* = 15.39; *p* < .001), although MP and MG showed a much higher level of similarity (Figure S1A,B). Likewise, three of the five ant species identified through morphological taxonomy (with sufficient replicates) showed different chemical cue profiles (Figure S1C, PERMANOVA: *df* = 2; pseudo‐*F* = 4.88; *p* < .001).

### 3.5 Comparison between *C. linsenmairi* ants found in two different plant hosts species

The behavior of *C. linsemairi* differed depending on the tree host. *C. linsenmairi* ants accepted termites more on MP than on MG (Figure 1; Table 3). In MP, *C. linsemairi* accepted more MP FB than in MG. Surprisingly for ants in MG, heterospecific origin coccids were accepted more often than homospecific origin coccids, whereas for MP ants, coccids that originated from other MP and MW trees were accepted less often than those originating from MG (Figure 1; Table 4). Larvae had a higher C/N ratio in MG plants in comparison to MP, but for workers, this pattern was reversed, with a higher C/N ratio for workers in MP (Figure S3A). Ants in MP had lower *δ13C* (corrected for FB) in both larvae and workers. Stable isotope analyses suggest that the proportion of food sources used by the ants (FB and insect prey) differed depending on the plant host (Figure S3C). N percentages were higher for *C. linsemairi* larvae and workers in MG. However, workers had a lower C percentage in MG compared to MP (Figure S3D).

## 4 DISCUSSION

Our behavioral and chemical study confirms that the ant species obligately associated with *Macaranga* myrmecophytes are highly specialized with respect to their nutritional preferences. FB from different host species are selected by ants based on their chemical profiles rather than their composition or nutritional value. Furthermore, the behavior toward surrogate herbivores and coccids is not dependent on the ant species or the quality of the alternative plant‐provided FB. Taken together, these results demonstrate that symbiotic ant dietary behavior may depend on the plant host. We suggest that some mechanisms may be operating to ensure partner fidelity, e.g. coercion by the host plant that ensures only certain food resources can be used by the ants.

### 4.1 Rejection/acceptance of FB

FB differed in quality between plant species, with MG FB having a significantly higher C/N ratio (lower N content), in comparison to those of MP and MW. FB with higher N‐content have been shown to be more attractive for ants (Heil et al., 1998). In *Crematogaster* sp. 8, which is exclusively found on MW, we found the highest rejection rates of heterospecific FB originating from MG and MP, in spite of FB from MP having similar nutritional quality and composition (C/N ratio of FB and proteomic analyses) to those of MW. MG and MP ants more frequently accepted FB from both MG and MP hosts, thus being less specialized with regards to FB. However, MG ants accepted FB of MG more frequently in comparison to MP ants. This lower acceptance by MP ants may be related to the lower food quality (in MG FB), as MG ants showed less behavioral differences for MP FB which had higher nitrogen content. But acceptance of FB by ants may not solely be based on nutritional quality but potentially also on composition and chemical cues found on the FB.

The two most phylogenetically related *Macaranga* species (MP and MG) were not the most similar in protein composition of FB (MP was closer to MW). The distinctness in MG FB protein composition and digestion times could potentially relate to the symbiotic ants' behavioral differences, as proteins may influence either quality or digestibility of FB. However, since a protein sequence database is not available for the genus *Macaranga* we cannot discuss this further. But familiarity with olfactory cues of the *Macaranga* host they reside on may also result in a higher acceptance of FB from the hosting species. This matches the chemical cue analysis where the most distinct chemical profiles were for the most exclusive interaction (ants on MW) for which rejection (of other FB) and acceptance (of their own plant) were the most pronounced. We also found in preliminary trials (not reported here) that altering the smell of the FB originating from the host plant by hexane dilution leads to the rejection of the FB (only tested once on each tree species as a control).

The overall differences in ant behavior and FB chemical profile similarity between the plant species are in concordance with their phylogenetic relatedness (Bänfer et al., 2004), perhaps indicating recent diversification of the chemical signatures of the FB and the ant preference for these profiles. This is concordant with our predictions that the most exclusive systems (*Crematogaster* sp. 8 on MW) have the most restricted partner choice mechanisms. Olfactory cues have already been demonstrated to be important for the colonizing *Crematogaster* queen to recognize the right *Macaranga* plant host species (Inui et al., 2001). We suggest that even worker ants may reject plant food material with the wrong smell, implying that chemical cues play an important role not only in partner choice during colonization (Jürgens et al., 2006) but also in nutrient recognition. However, the only previous study to have tested this found no difference in acceptance rates of heterospecific and homospecific FB; only when offered FB from a different genus did ants reject them (Fiala & Maschwitz, 1990). These conflicting results could result from the ants being starved in a laboratory in contrast to our study that was conducted in the field.

### 4.2 Rejection/Acceptance of surrogate herbivores and coccids

The only previous study presenting different “foreign” food items observed much lower rates of acceptance (Fiala & Maschwitz, 1990) for *C. borneensis* on *M. triloba* (7% acceptance rate). This ant species was also found in some of our trees (*N* = 5) and accepted higher rates of termites as a food source (30% acceptance rate). Behavioral differences in the results could be due to the variety of food items used in that study (butter and fish) which were offered with no standard replication, but rather were used to explore the full ant dietary spectrum and not the ants' capacity to assimilate a standardized prey.

Ants on both MW and MG showed similar behavior in having high levels of rejection of the surrogate herbivore (termites) as a food source and to a lesser degree toward coccids. In contrast, MP ants showed much higher rates of acceptance. As acceptance rate of coccids from other plants was also generally higher in ants on MP, it seems that, in general, ants on MP have lower rates of rejection for food items not provided directly by the host plant. However, this trend differs for *C. linsenmairi* where ants in the MG host accept more MP and MW coccids than in the MP host (in MP, *C. linsenmairi* only accept more coccids from MG host). This could be related to MG coccids differing (different species and/or due to coccids chemical cues deriving from the host plant) and also to the way coccids may contribute differently to the diet and needs of the ants (carbohydrates vs. protein consumption).

### 4.3 The relation between behavior and trophic enrichment

MW workers, larvae, and coccids showed approximately a 2.5 increase in trophic enrichment (compared to the *δ15N* FB) which is coherent with FB being the main source of nitrogen for the ants. This result is supported by ant rejection of herbivores and the major contribution of FB for both larvae and workers indicated by *δ15N* (compared to coccids). Moreover, the higher C% in MW ant workers compared to MG ant workers, despite MW FB having lower C%, suggests that these ants mostly tend coccids for their honeydew.

MG ant and larva trophic enrichment indicates that coccids rather than FB are the food source (~2.4 increase in *δ15N* compared to coccids). Because MG FB have the poorest nitrogen content, it would make sense that the ants compensate by the consumption of coccids. This is concordant with model results of the contributions of the two different potential food sources and the behavioral experiment showing a higher rate of acceptance of coccids than termites. However, our finding contrasts with the conclusion that coccids are not preyed upon (Heckroth et al., 2001). This previous study was a pioneering attempt to disentangle the tripartite system and the role of the coccids; the authors concluded that coccids were not consumed when brought inside. We believe that the behavior of the symbiotic ants could be very different if the plant has been damaged previously (field extraction and domatia exposed) and if only a few workers are introduced with no queen or brood—the method used by Heckroth et al. (2001).

MP larvae had a higher trophic enrichment in comparison to FB and coccids and MP workers had higher trophic enrichment compared to coccids only. In both cases, the increase was found to be low (<1.5), particularly in comparison to their FB, which could therefore not be the main source of food. Despite MP FB having higher N content than MG FB, MP workers are the ants that have a much lower N content than the other two species. Taken together our results suggest that MP FB are not contributing as much in providing nutrition to MP ant colonies, particularly the workers (although FB were abundant on the selected plants). This could explain the broader diet breadth found in our behavioral experiment for MP ants, which accept more herbivores, coccids, and foreign FB, thus indicating potential consumption of alternative food sources with a lower *δ15N* ratio than the FB. For instance, specific nematodes (Maschwitz et al., 2016) and a fungus (Voglmayr et al., 2011) are found living in most *Macaranga* domatia and may also contribute to the trophic enrichment of ants in MP, although it is currently unknown whether the ants feed on them.

### 4.4 Potential plant host coercion

We demonstrate here that acceptance of food items varies between plant host species, which adds further complexity in understanding the mechanisms maintaining these mutualisms. Above we discuss the possibility that the ants may not necessarily consume only FB but also coccids, herbivores, or alternative undocumented food sources. However, ants of the same species (*C. linsenmairi*) were found to accept more insect herbivores on MP where FB are of higher quality and not when in association with MG where FB are of lower quality (C/N ratio, N percentage). Moreover, *C. linsenmairi* ants did not respond to coccids in the same way depending on the plant host. Although we do not have direct evidence for host plant coercion as observed in other ant–plant symbioses (Heil et al., 2014), the fact that *C. linsemairi* ants display different behavior across plant host species with respect to acceptance or rejection of food resources is intriguing. Such context‐dependent variation in ant dietary patterns across plant species has been documented elsewhere (Orona‐Tamayo et al., 2013). One possible mechanism for host plants to prevent energy intake from prey or foreign FB would be to increase the dependency of the ants on food provided by the host plant. If the plant is able to prevent the ants from consuming any alternative food source, then the plant will have much greater control over ant activity on the plant. The fact that the herbivores on the plant are rejected is potentially a by‐product of this mechanism. However, ants do accept to varying degrees symbiotic coccids from other plant species, indicating that any such coercion by plant hosts is not absolute. Considering that trophobiosis could be an evolutionary driver of myrmecophytism in many tree species around the world (Nelsen et al., 2018), it could be that specific chemical cues present on the scale insects (just like the FB) and absent from other herbivores are key to being accepted into domatia—to be raised for honeydew or consumption.

## 5 CONCLUSION

This study confirms that symbiotic *Crematogaster* ants often refuse to consume prey that they have spent energy attacking and that they may also reject symbiotic coccids. The rejection of FB is not linked to their quality or composition, but rather to their surface chemical cues, with acceptance being greater on plant hosts with broader ant partner diversity. We also demonstrated for the first time that, depending on the ant species and plant host species, these ants vary between being primary and secondary consumers, and that it is unlikely that any of these ant species has a highly specialized primary consumer digestive system. In addition to the ecological and evolutionary relevance of our findings, the differing behavior of *C. linsemairi* across different tree host species, combined with the isotope and proteomic results, shows some important differences in the *M. glandibracteolata–Crematogaster* system indicating potential host coercion. However, exploring this further would require proteomic analyses based on the genotype of these plant species, which was beyond the scope of this study. We envisage that in the future, a full protein database would allow analyses of differences between plant species in specific FB proteins, potentially revealing the protease inhibitors responsibly for host coercion. In addition, further ecological studies should conduct DNA screening of gut content of these symbiotic ants to further explore potential coccid consumption and chemical profile comparison of specialized symbiotic coccids and generalist ones. We also suggest to explore the consumption of yet other symbionts likes nematodes or even black yeast, about which little is known despite their confirmed presence within many multipartite obligate myrmecophytic systems in tropical rainforests on different continents (Blatrix et al., 2009; Maschwitz et al., 2016; Mayer et al., 2014; Voglmayr et al., 2011).

## AUTHOR CONTRIBUTIONS

**Mickal Y. I. Houadria:** Conceptualization (equal); data curation (equal); formal analysis (equal); funding acquisition (equal); investigation (equal); methodology (equal); writing – original draft (equal); writing – review and editing (equal). **Tom M. Fayle:** Funding acquisition (equal); writing – original draft (supporting); writing – review and editing (supporting). **Giulio Barone:** Data curation (equal); methodology (equal). **Thomas Schmitt:** Data curation (supporting); formal analysis (supporting); software (supporting). **Petr Konik:** Data curation (supporting); formal analysis (supporting); methodology (equal); visualization (supporting). **Heike Feldhaar:** Conceptualization (equal); funding acquisition (equal); project administration (equal); writing – original draft (equal); writing – review and editing (equal).

## CONFLICT OF INTEREST

The authors declare that there are no conflicts of interest.

## Supporting information


Appendix S1‐S3

## Data Availability

All data will be accessible online: https://datadryad.org/stash/share/S0TCOZDJHPN5iWRV‐w53PWN7mK0uYASxOVIjUDovQWQ.

## References

[ece39760-bib-0001] Bänfer, G. , Fiala, B. , & Weising, K. (2004). AFLP analysis of phylogenetic relationships among myrmecophytic species of *macaranga* (Euphorbiaceae) and their allies. Plant Systematics and Evolution, 249(3–4), 213–231. 10.1007/s00606-004-0219-4 11399144

[ece39760-bib-0002] Bidartondo, M. I. , Burghardt, B. , Gebauer, G. , Bruns, T. D. , & Read, D. J. (2004). Changing partners in the dark: Isotopic and molecular evidence of ectomycorrhizal liaisons between forest orchids and trees. Proceedings of the Royal Society B: Biological Sciences, 271(1550), 1799–1806. 10.1098/rspb.2004.2807 PMC1691795 15315895

[ece39760-bib-0003] Blatrix, R. , Bouamer, S. , Morand, S. , & Selosse, M. A. (2009). Ant‐plant mutualisms should be viewed as symbiotic communities. Plant Signaling and Behavior, 4(6), 554–556. 10.4161/psb.4.6.8733 19816123 PMC2688311

[ece39760-bib-0004] Christensen, R. H. B. (2015). Package ‘ordinal’.

[ece39760-bib-0005] Clarke, K. R. , & Gorley, R. (2006). Primer v6: User manual/tutorial. Primer‐E L.

[ece39760-bib-0006] Davidson, D. W. , Cook, S. C. , Snelling, R. R. , & Chua, T. H. (2003). Explaining the abundance of ants in lowland tropical rainforest canopies. Science, 300(5621), 969–972. 10.1126/science.1082074 12738862

[ece39760-bib-0007] Davies, S. T. J. D. (2001). Systematics of macaranga secsts. Pachystemon and Pruinosae (Euphorbaicae). Harvard Papers in Botany, 6(2), 371–448.

[ece39760-bib-0008] Eck, G. , Fiala, B. , Linsenmair, K. E. , Bin Hashim, R. , & Proksch, P. (2001). Trade‐off between chemical and biotic antiherbivore defense in the south east Asian plant genus *macaranga* . Journal of Chemical Ecology, 27(10), 1979–1996. 10.1023/A:1012234702403 11710606

[ece39760-bib-0009] Ewers, R. M. , Didham, R. , Fahrig, L. , Ferraz, G. , Hector, A. , Holt, R. D. , Kapos, V. , Reynolds, G. , Sinun, W. , Snaddon, J. L. , & Turner, E. C. (2011). A large‐scale forest fragmentation experiment: The stability of altered forest ecosystems project. Philosophical Transactions of the Royal Society B: Biological Sciences, 366(1582), 3292–3302. 10.1098/rstb.2011.0049 PMC3179633 22006969

[ece39760-bib-0010] Federle, W. , Maschwitz, U. , Fiala, B. , Riederer, M. , & Hölldobler, B. (1997). Slippery ant‐plants and skilful climbers: Selection and protection of specific ant partners by epicuticular wax blooms in *macaranga* (euphorbiaceae). Oecologia, 112(2), 217–224. 10.1007/s004420050303 28307573

[ece39760-bib-0011] Federle, W. , Maschwitz, U. , & Hölldobler, B. (2002). Pruning of host plant neighbours as defence against enemy ant invasions: *Crematogaster* ant partners of *macaranga* protected by “wax barriers” prune less than their congeners. Oecologia, 132(2), 264–270. 10.1007/s00442-002-0947-z 28547361

[ece39760-bib-0012] Feldhaar, H. , Gebauer, G. , & Blüthgen, N. (2010). Stable isotopes: Past and future in exposing secrets of ant nutrition (Hymenoptera: Formicidae). Myrmecol News, 13, 3–13.

[ece39760-bib-0013] Feldhaar, H. , Maschwitz, U. , & Fiala, B. (2016). Taxonomic revision of the obligate plant‐ants of the genus *Crematogaster* Lund (Hymenoptera: Formicidae: Myrmicinae), associated with *macaranga* Thouars (Euphorbiaceae) on Borneo and the Malay peninsula. Sociobiology, 63(1), 651–681. 10.13102/sociobiology.v63i1.949

[ece39760-bib-0014] Feldhaar, H. , Straka, J. , Krischke, M. , Berthold, K. , Stoll, S. , Mueller, M. J. , & Gross, R. (2007). Nutritional upgrading for omnivorous carpenter ants by the endosymbiont *Blochmannia* . BMC Biology, 5, 1–11. 10.1186/1741-7007-5-48 17971224 PMC2206011

[ece39760-bib-0015] Ferriere, R. , Bronstein, J. L. , Rinaldi, S. , Law, R. , & Gauduchon, M. (2002). Cheating and the evolutionary stability of mutualisms. Proceedings of the Royal Society B: Biological Sciences, 269(1493), 773–780. 10.1098/rspb.2001.1900 PMC1690960 11958708

[ece39760-bib-0016] Fiala, B. , Jakob, A. , & Maschwitz, U. (1999). Diversity, evolutionary specialization and geographic distribution of a mutualistic ant‐plant complex: *Macaranga* and *Crematogaster* in South East Asia. Biological Journal of the Linnean Society, 66, 305–331. 10.1111/j.1095-8312.1999.tb01893.x

[ece39760-bib-0017] Fiala, B. , & Maschwitz, U. (1990). Studies on the south east Asian ant‐plant association *Crematogaster borneensis/macaranga*: Adaptations of the ant partner. Insectes Sociaux, 37(1), 212–231.

[ece39760-bib-0018] Fiala, B. , & Maschwitz, U. (1992). Food bodies and their significance for obligate ant‐association in the tree genus *macaranga* (Euphorbiaceae). Botanical Journal of the Linnean Society, 110, 61–75.

[ece39760-bib-0019] Frederickson, M. E. (2017). Mutualisms are not on the verge of breakdown. Trends in Ecology and Evolution, 32(10), 727–734. 10.1016/j.tree.2017.07.001 28739078

[ece39760-bib-0020] Giron, D. , Dubreuil, G. , Bennett, A. , Dedeine, F. , Dicke, M. , Dyer, L. A. , Erb, M. , Harris, M. , Huguet, E. , Kaloshian, I. , Kawakita, A. , Lopez Vaamonde, C. , Palmer, T. , Petanidou, T. , Poulsen, M. , Sallé, A. , Simon, J. C. , Terblanche, J. , Thiery, D. , & Pincebourde, S. (2018). Promises and challenges in insect–plant interactions. Entomologia Experimentalis et Applicata, 166(5), 319–343. 10.1111/eea.12679

[ece39760-bib-0021] Grasso, D. A. , Pandolfi, C. , Bazihizina, N. , Nocentini, D. , Nepi, M. , & Mancuso, S. (2015). Extrafloral‐nectar‐based partner manipulation in plant‐ant relationships. AoB PLANTS, 7(1), 1–15. 10.1093/aobpla/plv002 PMC4326690 25589521

[ece39760-bib-0022] Heckroth, H. P. , Fiala, B. , Gullan, P. J. , Idris, A. H. , & Maschwitz, U. (1998). The soft scale (Coccidae) associates of Malaysian ant‐plants. Journal of Tropical Ecology, 14(4), 427–443. 10.1017/S0266467498000327

[ece39760-bib-0023] Heckroth, H. P. , Fiala, B. , & Maschwitz, U. (2001). Integration of scale insects (Hemiptera: Coccidae) in the south‐east Asian ant‐plant (*Crematogaster* (Formicidae)‐*macaranga* (Euphorbiacae)) system. Entomologica, 33, 287–295.

[ece39760-bib-0024] Heil, M. , Barajas‐Barron, A. , Orona‐Tamayo, D. , Wielsch, N. , & Svatos, A. (2014). Partner manipulation stabilises a horizontally transmitted mutualism. Ecology Letters, 17(2), 185–192. 10.1111/ele.12215 24188323

[ece39760-bib-0025] Heil, M. , Fiala, B. , Kaiser, W. M. , & Linsenmair, K. E. (1998). Chemical contents of *macaranga* food bodies: Adaptations to their role in ant attraction and nutrition. Functional Ecology, 12(1), 117–122. 10.1046/j.1365-2435.1998.00158.x

[ece39760-bib-0026] Heil, M. , Fiala, B. , Maschwitz, U. , & Linsenmair, K. E. (2001). On benefits of indirect defence: Short‐ and long‐term studies of antiherbivore protection via mutualistic ants. Oecologia, 126(3), 395–403. 10.1007/s004420000532 28547454

[ece39760-bib-0027] Heil, M. , Gonzalez‐Teuber, M. , Clement, L. W. , Kautz, S. , Verhaagh, M. , & Bueno, J. C. S. (2009). Divergent investment strategies of acacia myrmecophytes and the coexistence of mutalists and exploiters. Proceedings of the National Academy of Sciences, 106(43), 18091–18096. 10.1073/pnas.0904304106 PMC2775331 19717429

[ece39760-bib-0028] Heil, M. , Hilpert, A. , Fiala, B. , & Eduard Linsenmair, K. (2001). Nutrient availability and indirect (biotic) defence in a Malaysian ant‐plant. Oecologia, 126(3), 404–408. 10.1007/s004420000534 28547455

[ece39760-bib-0029] Inui, Y. , Itioka, T. , Murase, K. , & Yamaoka, R. (2001). Chemical recognition of partner plant species by foundress ant queens in *macaranga‐Crematogaster* myrmecophytism. Journal of Chemical Ecology, 27(10), 2029–2040. 10.1023/A:1012290820150 11710609

[ece39760-bib-0030] Itino, T. , Itioka, T. , Hatada, A. , & Hamid, A. A. (2001). Effects of food rewards offered by ant‐plant *macaranga* on the colony size of ants. Ecological Research, 16(4), 775–786. 10.1046/j.1440-1703.2001.00433.x

[ece39760-bib-0031] Jürgens, A. , Feldhaar, H. , Feldmeyer, B. , & Fiala, B. (2006). Chemical composition of leaf volatiles in *macaranga* species (Euphorbiaceae) and their potential role as olfactory cues in host‐localization of foundress queens of specific ant partners. Biochemical Systematics and Ecology, 34(2), 97–113. 10.1016/j.bse.2005.08.005

[ece39760-bib-0032] Lüdecke, D. (2018). Ggeffects: Tidy data frames of marginal effects from regression models. Journal of Open Source Software, 3(26), 772. 10.21105/joss.00772

[ece39760-bib-0033] Maschwitz, U. , Fiala, B. , Dumpert, K. , Hashim, R. , & Sudhaus, W. (2016). Nematode associates and bacteria in ant‐tree symbioses. Symbiosis, 69, 1–7. 10.1007/s13199-015-0367-6

[ece39760-bib-0034] Maschwitz, U. , Fiala, B. , Lee, Y. F. , Chey, V. K. , & Tan, F. L. (1989). New and little‐known myrmecophytic associations from bornean rain forests. Malayan Nature Journal, 43, 106–115.

[ece39760-bib-0035] Mayer, V. , Frederickson, M. E. , McKey, D. , & Blatrix, R. (2014). Current issues in the evolutionary ecology of ant‐plant symbioses. New Phytologist, 202(3), 749–764. 10.1111/nph.12690 24444030

[ece39760-bib-0036] Moog, J. (2009). The associations of the plant‐ant Cladomyrma with plants in Southeast Asia. Fachbereich Biowissenschaf Ten der Johann Wolfgang Goethe‐Universität, 2, 17–32.

[ece39760-bib-0037] Moog, J. , Saw, L. G. , Hashim, R. , & Maschwitz, U. (2005). The triple alliance: How a plant‐ant, living in an ant‐plant, acquires the third partner, a scale insect. Insectes Sociaux, 52(2), 169–176. 10.1007/s00040-005-0791-3

[ece39760-bib-0038] Nelsen, M. P. , Ree, R. H. , & Moreau, C. S. (2018). Ant–plant interactions evolved through increasing interdependence. Proceedings of the National Academy of Sciences, 115(48), 12253–12258. 10.1073/pnas.1719794115 PMC6275543 30420513

[ece39760-bib-0039] Nepi, M. , Grasso, D. A. , & Mancuso, S. (2018). Nectar in plant–insect mutualistic relationships: From food reward to partner manipulation. Frontiers in Plant Science, 9(1063), 1–14. 10.3389/fpls.2018.01063 30073014 PMC6060274

[ece39760-bib-0040] Orona‐Tamayo, D. , & Heil, M. (2013). Stabilizing mutualisms threatened by exploiters: New insights from ant‐plant research. Biotropica, 45(6), 654–665. 10.1111/btp.12059

[ece39760-bib-0041] Orona‐Tamayo, D. , Wielsch, N. , Blanco‐Labra, A. , Svatos, A. , Farías‐Rodríguez, R. , & Heil, M. (2013). Exclusive rewards in: Ant proteases and plant protease inhibitors create a lock‐key system to protect acacia food bodies from exploitation. Molecular Ecology, 22(15), 4087–4100. 10.1111/mec.12320 23683294

[ece39760-bib-0042] Parnell, A. C. , Phillips, D. L. , Bearhop, S. , Semmens, B. X. , Ward, E. J. , Moore, J. W. , Jacksong, A. L. , Greyh, J. , Kellyg, D. J. , & Inger, R. (2013). Bayesian stable isotope mixing models. Environmetrics, 24(6), 387–399. 10.1002/env.2221

[ece39760-bib-0043] Ribeiro, L. F. , Solar, R. R. C. , Sobrinho, T. G. , Muscardi, D. C. , Schoereder, J. H. , & Andersen, A. N. (2019). Different trophic groups of arboreal ants show differential responses to resource supplementation in a neotropical savanna. Oecologia, 190(2), 433–443. 10.1007/s00442-019-04414-z 31069514

[ece39760-bib-0044] Sagers, L. , Ginger, M. , & Evans, D. (2000). Carbon and nitrogen isotopes trace nutrient exchange in an ant‐plant mutualism. Oecologia, 123, 582–586. 28308767 10.1007/PL00008863

[ece39760-bib-0045] Souto‐Vilarós, D. , Proffit, M. , Buatois, B. , Rindos, M. , Sisol, M. , Kuyaiva, T. , Isua, B. , Michalek, J. , Darwell, C. , Hossaert‐McKey, M. , Weiblen, G. , Novotny, V. , & Segar, S. T. (2018). Pollination along an elevational gradient mediated both by floral scent and pollinator compatibility in the fig and fig‐wasp mutualism. Journal of Ecology, 106(6), 2256–2273. 10.1111/1365-2745.12995

[ece39760-bib-0046] Ueda, S. , Quek, S. P. , Itioka, T. , & Murase, K. (2010). Phylogeography of the *Coccus* scale insects inhabiting myrmecophytic *macaranga* plants in Southeast Asia. Population Ecology, 52(1), 137–146. 10.1007/s10144-009-0162-4

[ece39760-bib-0047] van Welzen, P. C. , Strijk, J. S. , van Konijnenburg‐van Cittert, J. H. , Nucete, M. , & Merckx, V. S. F. T. (2014). Dated phylogenies of the sister genera Macaranga and Mallotus (Euphorbiaceae): congruence in historical biogeographic patterns? PLoS One, 9(1), 1–14. 10.1371/journal.pone.0085713 PMC3894986 24465660

[ece39760-bib-0048] Voglmayr, H. , Mayer, V. , Maschwitz, U. , & Moog, J. (2011). The diversity of ant‐associated black yeasts: Insights into a newly discovered world of symbiotic interactions. British Mycological Society, 5(115), 1077–1091. 10.1016/j.funbio.2010.11.006 21944219

[ece39760-bib-0049] Wickham, H. , Averick, M. , Bryan, J. , Chang, W. , McGowan, L. , François, R. , Grolemund, G. , Hayes, A. , Henry, L. , Hester, J. , Kuhn, M. , Pedersen, T. L. , Miller, E. , Bache, S. M. , Müller, K. , Ooms, J. , Robinson, D. , Seidel, D. P. , Spinu, V. , Takahashi, K. , Vaughan, D. , Wilke, C. , Woo, K. , & Yutani, H. (2019). Welcome to the Tidyverse. Journal of Open Source Software, 4(43), 1686. 10.21105/joss.01686

