# Supplementary material for: An experimental, behavioral, and chemical analysis of food limitations in mutualistic Crematogaster ant symbionts inhabiting Macaranga host plants
Source: Ecol Evol. 2023 Feb 7;13(2):e9760. doi: 10.1002/ece3.9760 (PMC9905419; doi:10.1002/ece3.9760)
Supplement: Supplementary file 1 — Appendix S1‐S3 [file ECE3-13-e9760-s001.docx]

**Supplementary Information**

**Appendix 1:** Isotopic analyses

Measured relative isotope abundances are denoted as δ values that were calculated according to the following equation: δ13C or δ15N, = (Rsample/Rstandard − 1) × 1000 (‰), where Rsample and Rstandard are the ratios of heavy to light isotope of the samples and the respective standard. Standard gases (Riessner, Lichtenfels, Germany) were calibrated with respect to international standards (CO2 vs. PDB, N2 vs. N2 in air) with the reference substances ANU sucrose and NBS19 for the C isotopes, N1 and N2 for the N isotopes all provided by the IAEA (International Atomic Energy Agency, Vienna, Austria). Reproducibility and accuracy of the C and N isotope abundance measurements were routinely controlled by measuring the laboratory standard acetanilide (Gebauer and Schulze, 1991). In relative C and N isotope natural abundance analyses, acetanilide was routinely analysed at least six times per batch of 50 samples. The maximum variation in δ13C and δ15N both within and between batches was always below 0.2‰. Total N concentrations of the samples were calculated from sample weights and peak areas using a six-point calibration curve per sample run based on measurements of the laboratory standard acetanilide with a known N concentration of 10.36% and C concentrations of 10.36% and 71.09%, respectively (Gebauer and Schulze, 1991).

**Appendix 2:** Chemical cues analyses

he GC (split/splitless injector in splitless mode for 1 min, injected volume 1 μl at 300°C) was equipped with a DB-5 Fused Silica capillary column (30 m x 0.25 mm ID, df = 0.25 µm; J&W Scientific, Folsom, USA). Helium served as carrier gas at a constant flow of 1 ml/min. The following temperature program was used: Start temperature 60°C, temperature increase by 5°C per min up to 300 °C, isotherm at 300°C for 10 min. The electron ionization mass spectra (EI-MS) were acquired at an ionization voltage of 70 eV (source temperature: 230°C). Chromatograms and mass spectra were recorded and quantified via integrated peak areas with the software HP Enhanced ChemStation G1701AA (version A.03.00; Hewlett Packard). CHC compounds were identified by the compound specific retention indices and their diagnostic ions (Carlson et al. 1998).

**Appendix 3:** Proteomic analyses

To produce the 30 min digested sample, FB from all three plant species were placed in 30 µL of 100 mM ammonium bicarbonate (Sigma Aldrich), and sonicated by 10 pulses using a UP100H ultrasonic processor (Hielscher , Germany) on ice. After a brief centrifugation, proteomic grade trypsin (Sigma Aldrich) was added to the mixture and the test tube was incubated at 37 °C. After 30 min, the fluid was aspirated, fresh trypsin was added to it, and it was incubated overnight at 37 °C. The next day, fresh trypsin was added, the suspension and the FB were sonicated again the same way and incubated at 37°C. After 12h of incubation, fluid was again separated, labeled as 12h digest sample and set aside. Fresh trypsin was added to the FB, sonicated and incubated at 37°C for another 12h to become the 24h digest sample. The procedure was repeated once more to obtain the 36 hour digest sample. The trypsin added to the FB for all the time points was at a concentration of 10ng/µL in 100mM ammonium bicarbonate. From the collected samples (30min, 12h, 24h and 36h), peptides were isolated using StageTips according to Rappsilber *et al*. 2007. The resulting 20µL of peptide mixture in 0.1% formic acid (Fluka) was then placed in a glass vial suited for the autosampler of the LC/MS machine.

Peptides were separated by reverse-phase UHPLC on a BEH300 C18 analytical column (75 mm i.d.; 150 mm length, particle size 1.7 mm; Waters) that was perfused at 0.4 µl/min flow rate with 0.1% formic acid containing acetonitrile in concentration gradually increasing from 3% (v/v) to 40% (v/v) in the course of 30 min. Peptides eluted from the column flowed directly into the nano-electrospray ionization source. Raw data for each sample was acquired in data-independent MS^E mode. Peptide and fragment spectra were acquired with 2 ppm and 5 ppm tolerance, respectively. PLGS 3.0 (Waters) and Progenesis (Nonlinear Dynamics) software was used for matching generated data with the entries in the species specific Uniprot and NCBI protein databases, and generating protein and peptide ion intensity data. Identification of 2-3 consecutive y- or b-ions was required for positive peptide match.^

| **Table S1** : Euclidean distances of FB composition depending on the digestion time and plant host. In bold red is the only time when MP and MW were closer to MG than to each other. | | | | |
| --- | --- | --- | --- | --- |
| Digestion time | Species | Average Distance between/within groups | | |
|  |  | MG | MP | MW |
| 30min | MG | 4.40 |  |  |
|  | MP | 7.61 | 4.28 |  |
|  | MW | 7.83 | 5.49 | 3.08 |
| **12h** | MG | 1.16 |  |  |
|  | MP | **3.22** | 0.90 |  |
|  | MW | **3.28** | **3.40** | 0.73 |
| 24h | MG | 0.96 |  |  |
|  | MP | 3.62 | 1.06 |  |
|  | MW | 4.12 | 3.12 | 1.24 |
| 36h | MG | 2.83 |  |  |
|  | MP | 5.41 | 0.83 |  |
|  | MW | 6.14 | 2.94 | 2.19 |


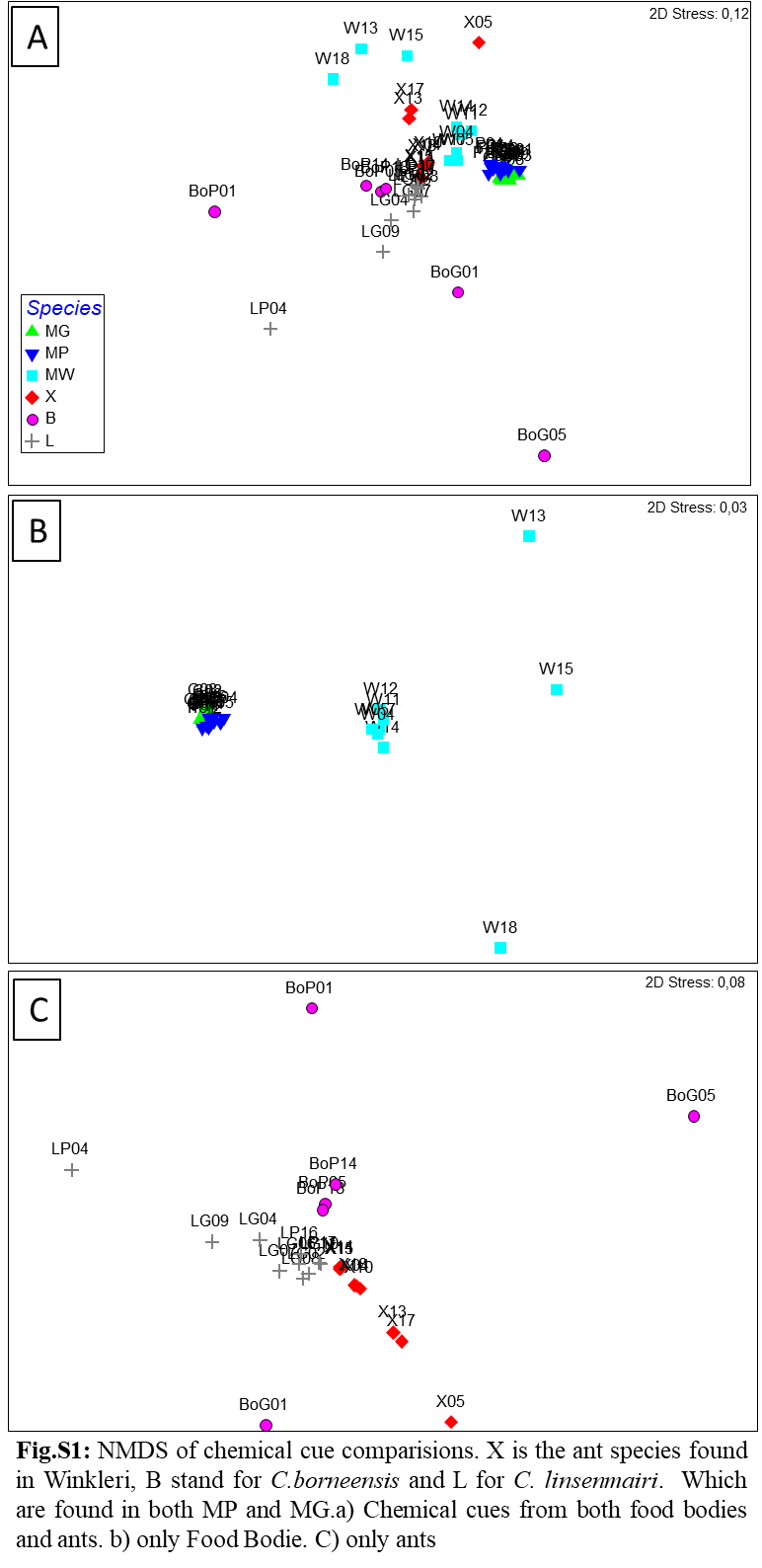


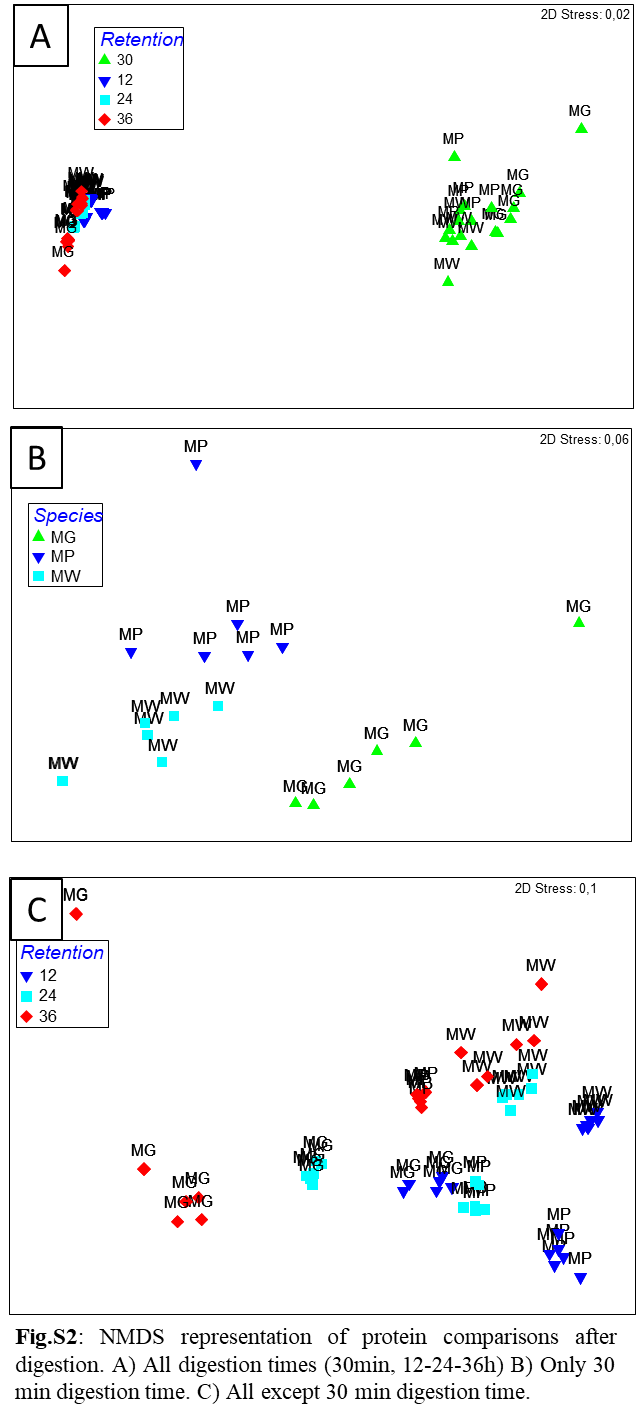


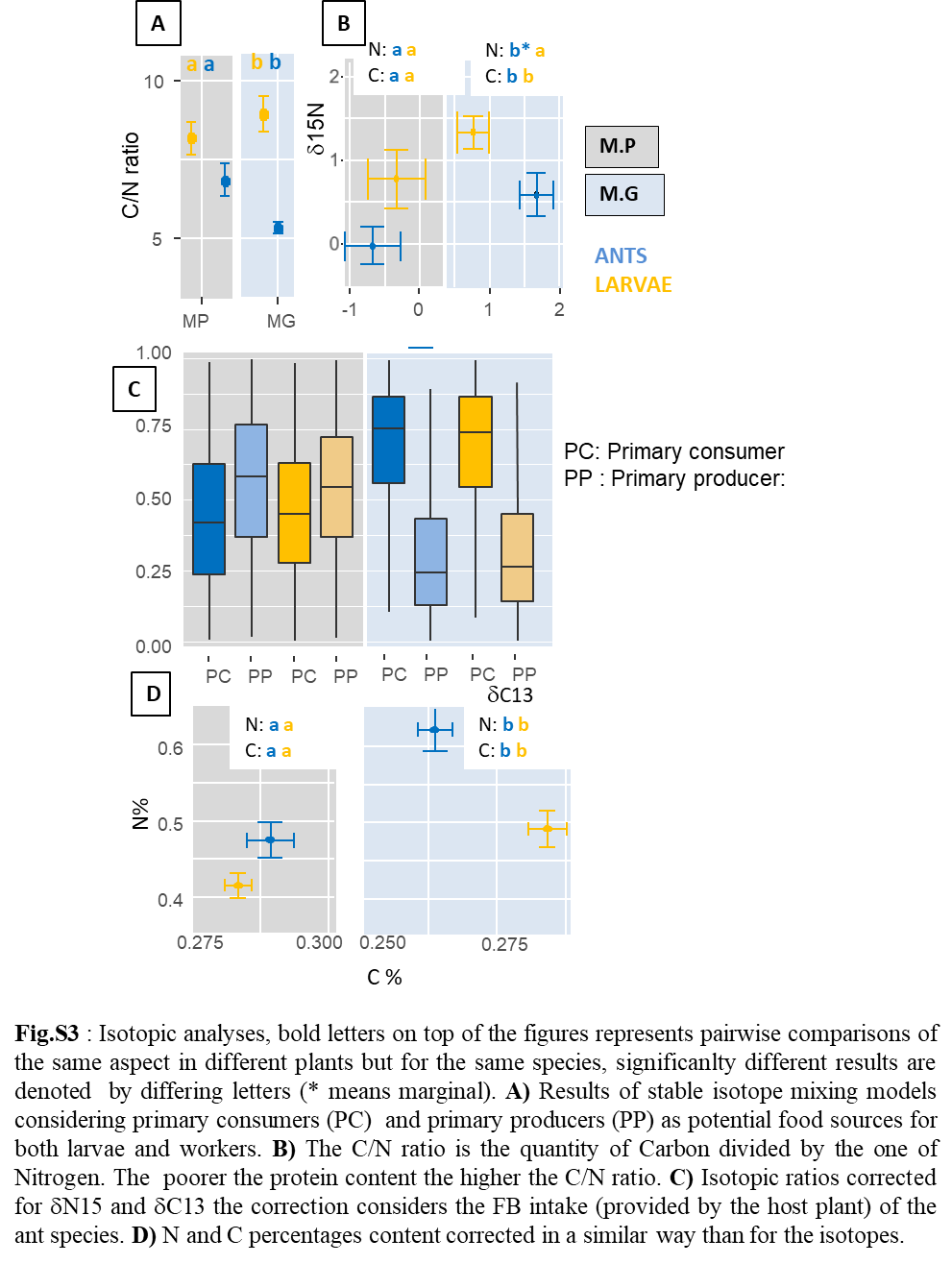


| **Table S2: Protein identification PLGS 3.0 (Waters) and Progenesis (Nonlinear Dynamics) software was used for matching generated data with the entries in the species specific Uniprot and NCBI protein databases, and generating protein and peptide ion intensity data. Identification of 2-3 consecutive y- or b-ions was required for positive peptide match** | | | |
| --- | --- | --- | --- |
| **Accession** | **Peptides** | **Unique peptides** | **Description** |
| **P02827** | **7** | **1** | **Heat shock 70 kDa protein (HSP70).** |
| **P14037** | **1** | **1** | **30S ribosomal protein S7P.** |
| **P21239;P08926** | **8** | **1** | **RuBisCO subunit binding-protein alpha subunit_ chl** |
| **Q9Y896** | **1** | **1** | **Actin 2.** |
| **Q08080** | **1** | **1** | **Stromal 70 kDa heat shock-related protein_ chlorop** |
| **Q56235** | **6** | **3** | **Chaperone protein dnaK (Heat shock protein 70) (He** |
| **P00761;P06871** | **2** | **1** | **Trypsin precursor (EC 3.4.21.4).** |
| **P31542** | **1** | **1** | **ATP-dependent clp protease ATP-binding subunit clp** |
| **O33528** | **3** | **2** | **Chaperone protein dnaK (Heat shock protein 70) (He** |
| **Q10657** | **1** | **1** | **Triosephosphate isomerase (EC 5.3.1.1) (TIM).** |
| **P03261;P04495** | **1** | **1** | **DNA polymerase (EC 2.7.7.7).** |
| **P50246;O23255;P32112;P35007;P50248;P50249;P93253;Q01781** | **2** | **1** | **Adenosylhomocysteinase (EC 3.3.1.1) (S-adenosyl-L-** |
| **Q03274** | **2** | **1** | **Retrovirus-related POL polyprotein from type I ret** |
| **P37282** | **3** | **3** | **60 kDa chaperonin (Protein Cpn60) (groEL protein).** |
| **Q9L7Z1** | **3** | **1** | **Chaperone protein dnaK (Heat shock protein 70) (He** |
| **P43722** | **1** | **1** | **DNA-binding protein HU.** |
| **P14963** | **6** | **2** | **Elongation factor 1-alpha (EF-1-alpha).** |
| **P27179** | **1** | **1** | **ATP synthase alpha chain (EC 3.6.3.14).** |
| **P24629;P16121** | **18** | **4** | **Heat shock cognate 70 kDa protein 1.** |
| **P40918** | **6** | **2** | **Heat shock 70 kDa protein (Allergen Cla h 4) (Cla** |
| **O05700** | **3** | **1** | **Chaperone protein dnaK (Heat shock protein 70) (He** |
| **P39207;P47919;P47920;Q39839;Q96559** | **3** | **2** | **Nucleoside diphosphate kinase I (EC 2.7.4.6) (NDK** |
| **Q9L7Q5** | **1** | **1** | **DNA gyrase subunit A (EC 5.99.1.3).** |
| **P57588** | **1** | **1** | **50S ribosomal protein L2.** |
| **P42645;** | **4** | **1** | **14-3-3-like protein GF14 upsilon (General regulato** |
| **Q12272** | **1** | **1** | **tRNA 2'phosphotransferase (EC 2.7.-.-).** |
| **P02515** | **1** | **1** | **Heat shock protein 22.** |
| **P11146** | **8** | **2** | **Heat shock 70 kDa protein cognate 2 (Heat shock 70** |
| **P41753** | **8** | **3** | **Heat shock 70 kDa protein.** |
| **P13535** | **5** | **1** | **Myosin heavy chain_ skeletal muscle_ perinatal (My** |
| **P41825;P41826;P41827** | **8** | **4** | **Heat shock protein 70 A1.** |
| **Q07437;P14834** | **10** | **3** | **Heat shock 70 kDa protein.** |
| **Q9LKR3;Q39043;Q03685** | **12** | **3** | **Luminal binding protein 1 precursor (BiP1) (AtBP1)** |
| **P21240;P21241** | **6** | **1** | **RuBisCO subunit binding-protein beta subunit_ chlo** |
| **P01375** | **1** | **1** | **Tumor necrosis factor precursor (TNF-alpha) (Cache** |
| **Q9PJR8** | **1** | **1** | **Phenylalanyl-tRNA synthetase beta chain (EC 6.1.1.** |
| **P19107** | **2** | **1** | **Phosrestin I (Arrestin B) (Arrestin 2) (49 kDa arr** |
| **Q9N599** | **1** | **1** | **Probable proteasome subunit alpha type 4 (EC 3.4.2** |
| **P11143;P81672;P87047;Q52701;Q55154;Q92260** | **20** | **4** | **Heat shock 70 kDa protein.** |
| **P77819** | **3** | **1** | **DNA-directed RNA polymerase beta' chain (EC 2.7.7.** |
| **P51059;P29194** | **3** | **1** | **Phosphoenolpyruvate carboxylase 2 (EC 4.1.1.31) (P** |
| **P16394** | **8** | **1** | **Heat shock 70 kDa protein.** |
| **Q42667** | **2** | **1** | **Phenylalanine ammonia-lyase (EC 4.3.1.5).** |
| **P90519** | **5** | **1** | **Elongation factor 1-alpha (EF-1-alpha).** |
| **P04970;P24753** | **3** | **2** | **Glyceraldehyde 3-phosphate dehydrogenase 1 (EC 1.2** |
| **Q02986** | **1** | **1** | **Imidazoleglycerol-phosphate dehydratase (EC 4.2.1.** |
| **P21238** | **7** | **1** | **RuBisCO subunit binding-protein alpha subunit_ chl** |
| **P56255** | **2** | **1** | **ATP-dependent DNA helicase pcrA (EC 3.6.1.-).** |
| **P42895** | **3** | **1** | **Enolase 2 (EC 4.2.1.11) (2-phosphoglycerate dehydr** |
| **Q9ZDA3** | **1** | **1** | **Hypothetical protein RP439.** |
| **P46561** | **2** | **1** | **Probable ATP synthase beta chain_ mitochondrial pr** |
| **P25861;P04796;P34783;P34921;Q42671;P09094;Q43247;P26518;P26520;Q39769;Q41595;P08735;P22513;P26517;P52694;Q09054;Q27890;Q42977;Q94469** | **8** | **3** | **Glyceraldehyde 3-phosphate dehydrogenase_ cytosoli** |
| **P41719** | **2** | **1** | **Serine/threonine-protein kinase 1 (EC 2.7.1.-).** |
| **P22954** | **12** | **2** | **Heat shock cognate 70 kDa protein 2 (Hsc70.2).** |
| **P22202** | **10** | **1** | **Heat shock protein SSA4.** |
| **Q08277;P51819** | **3** | **1** | **Heat shock protein 82.** |
| **P20030** | **7** | **3** | **Heat shock cognate HSP70 protein.** |
| **P34922** | **5** | **1** | **Glyceraldehyde 3-phosphate dehydrogenase_ cytosoli** |
| **P36181;P51818;P33126** | **6** | **3** | **Heat shock cognate protein 80.** |
| **Q16956** | **7** | **2** | **78 kDa glucose-regulated protein precursor (GRP 78** |
| **Q60821** | **1** | **1** | **Zinc finger protein 151 (Polyomavirus late initiat** |
| **P51469** | **1** | **1** | **Glyceraldehyde 3-phosphate dehydrogenase** |
| **O31219** | **2** | **1** | **Aspartate-semialdehyde dehydrogenase (EC 1.2.1.11)** |
| **P10591;P10592** | **9** | **3** | **Heat shock protein SSA1 (Heat shock protein YG100)** |
| **O09486** | **1** | **1** | **S-adenosylmethionine synthetase (EC 2.5.1.6)** |
| **P02824;P02825;P82910** | **3** | **1** | **Major heat shock 70 kDa protein B (Heat shock prot** |
| **P93263;Q42699** | **3** | **1** | **5-methyltetrahydropteroyltriglutamate--homocystein** |
| **P09435;P48741** | **12** | **3** | **Heat shock protein SSA3.** |
| **Q42676** | **1** | **1** | **Transketolase_ chloroplast (EC 2.2.1.1) (TK) (Frag** |
| **O83949** | **3** | **1** | **Chaperone protein htpG (Heat shock protein htpG) (** |
| **P41633** | **1** | **1** | **Chloroplast 50S ribosomal protein L14.** |
| **P29843;P02826** | **11** | **1** | **Heat shock 70 kDa protein cognate 1 (Heat shock 70** |
| **P06588** | **1** | **1** | **Chloroplast 30S ribosomal protein S19.** |
| **P08418** | **11** | **2** | **Heat shock 70 kDa homolog protein (HSP70) (Major s** |
| **P16019** | **6** | **2** | **Heat shock 70 kDa protein (HSP 70.1).** |
| **P93254** | **1** | **1** | **S-adenosylmethionine synthetase (EC 2.5.1.6) (Meth** |
| **P03310** | **1** | **1** | **Genome polyprotein [Contains: Coat protein VP1; Co** |
| **P51183** | **1** | **1** | **Phosphoenolpyruvate-protein phosphotransferase (EC** |
| **P46257** | **3** | **1** | **Fructose-bisphosphate aldolase_ cytoplasmic isozym** |
| **Q9U639** | **8** | **1** | **Heat shock 70 kDa protein cognate 4.** |
| **O05272** | **1** | **1** | **Asparagine synthetase [glutamine-hydrolyzing] 3 (E** |
| **Q04967** | **6** | **1** | **Heat shock 70 kDa protein 6 (Heat shock 70 kDa pro** |
| **P29844;Q06248** | **11** | **3** | **Heat shock 70 kDa protein cognate 3 precursor (78** |
| **Q927Y3** | **1** | **1** | **Preprotein translocase secA subunit.** |
| **Q60287** | **2** | **1** | **Hypothetical protein MJECL28.** |
| **P36435** | **3** | **1** | **Probable intron maturase (Maturase K).** |
| **O34241** | **3** | **1** | **Chaperone protein dnaK (Heat shock protein 70) (He** |
| **Q00251;** | **8** | **2** | **Elongation factor 1-alpha (EF-1-alpha).** |
| **P26988** | **3** | **1** | **Glyceraldehyde 3-phosphate dehydrogenase (EC 1.2.1** |
| **P30167;P53494;P53497;P81228;P81229;P93371;Q05214;P30170** | **4** | **1** | **Actin 58.** |
| **P03427;P21428** | **1** | **1** | **RNA-directed RNA polymerase subunit P3 (EC 2.7.7.4** |
| **P54243** | **3** | **1** | **Glucose-6-phosphate isomerase_ cytosolic (EC 5.3.1** |
| **P47547** | **4** | **1** | **Chaperone protein dnaK (Heat shock protein 70) (He** |
| **P29845** | **4** | **1** | **Heat shock 70 kDa protein cognate 5.** |
| **P46871** | **1** | **1** | **Kinesin-II 95 kDa subunit (KRP-85/95 95 kDa subuni** |
| **P48313** | **3** | **2** | **DNA terminal protein (Bellett protein) (pTP protei** |
| **Q39613;P21569** | **4** | **1** | **Peptidyl-prolyl cis-trans isomerase (EC 5.2.1.8) (** |
| **Q42807** | **2** | **1** | **Acyl-[acyl-carrier protein] desaturase_ chloroplas** |
| **P08440** | **2** | **1** | **Fructose-bisphosphate aldolase_ cytoplasmic isozym** |
| **P54235;P54242** | **2** | **1** | **Glucose-6-phosphate isomerase_ cytosolic 1 (EC 5.3** |
| **P57139** | **1** | **1** | **Bifunctional glmU protein [Includes: UDP-N-acetylg** |
| **O60333** | **4** | **2** | **Kinesin-like protein KIF1B (Klp).** |
| **P73334;O78483;P22705** | **2** | **1** | **DNA-directed RNA polymerase delta chain (EC 2.7.7.** |
| **P53991** | **1** | **1** | **Ferredoxin--NADP reductase_ chloroplast precursor** |
| **P19976;P19975;P25699** | **4** | **2** | **Ferritin_ chloroplast precursor (SOF-35).** |
| **P56952** | **1** | **1** | **3-phosphoshikimate 1-carboxyvinyltransferase (EC 2** |
| **Q9ZAZ6** | **1** | **1** | **Pesticidial crystal protein cry1Gb (Insecticidal d** |
| **Q05944** | **3** | **1** | **Heat shock 70 kDa protein.** |
| **P52962** | **1** | **1** | **Moesin.** |
| **Q01233** | **6** | **2** | **Heat shock 70 kDa protein (HSP70).** |
| **Q01297;P25819;P25890;P48351;P48352;P49317;Q42547;P55308;P55310** | **3** | **1** | **Catalase isozyme 1 (EC 1.11.1.6).** |
| **Q05981** | **2** | **1** | **Chaperone protein dnaK (Heat shock protein 70) (He** |
| **P13540;P02564;P79293;P12883;P02562;P04462;P11778;P13542** | **7** | **1** | **Myosin heavy chain_ cardiac muscle beta isoform (M** |
| **P41148;P14625;Q29092;P08113;O18750;P08110;O33012;O44001;P02828;P04809;P04810;P04811;P08238;P11499;P34058;Q04619;Q50667;Q97E05;Q9ZMM2** | **8** | **5** | **Endoplasmin precursor (94 kDa glucose-regulated pr** |
| **Q9XD14** | **1** | **1** | **Translation initiation factor IF-1.** |
| **P42555** | **4** | **2** | **Chaperone protein htpG (Heat shock protein htpG) (** |
| **P43451;P95789** | **1** | **1** | **ATP synthase beta chain (EC 3.6.3.14).** |
| **O02654;P42894** | **1** | **1** | **Enolase (EC 4.2.1.11) (2-phosphoglycerate dehydrat** |
| **P41376;P35683;Q41741;P38919;P41377;P41378;P41379;P41381;P41382;Q40465;Q40466;Q40467;Q40468;Q40470;Q40471** | **3** | **1** | **Eukaryotic initiation factor 4A-1 (eIF-4A-1) (eIF4** |
| **P08106** | **9** | **3** | **Heat shock 70 kDa protein (HSP70).** |
| **Q05746** | **8** | **1** | **Heat shock 70 kDa protein (HSP70) (Cytoplasmic ant** |
| **Q01330** | **1** | **1** | **Zeaxanthin glucosyl transferase (EC 2.4.1.-).** |
| **Q9WYK6** | **2** | **1** | **Chaperone protein dnaK (Heat shock protein 70) (He** |
| **P39002** | **1** | **1** | **Long-chain-fatty-acid--CoA ligase 3 (EC 6.2.1.3) (** |
| **P39209** | **2** | **1** | **Methyl-accepting chemotaxis protein tlpC.** |
| **Q03878;Q99069** | **1** | **1** | **Glycine-rich RNA-binding protein.** |
| **Q62949** | **1** | **1** | **Cortistatin precursor [Contains: Cortistatin-29; C** |
| **P34924** | **2** | **1** | **Glyceraldehyde 3-phosphate dehydrogenase_ cytosoli** |
| **P13549;P04720;P10126;P20001;Q90835;P02993;P27706;Q05639** | **9** | **1** | **Elongation factor 1-alpha_ somatic form (EF-1-alph** |
| **Q9NR22** | **2** | **1** | **Protein arginine N-methyltransferase 4 (EC 2.1.1.-** |
| **Q60024** | **2** | **1** | **60 kDa chaperonin (Protein Cpn60) (groEL protein).** |
| **P56966;Q9WTN0** | **1** | **1** | **Geranylgeranyl pyrophosphate synthetase (GGPP synt** |
| **Q58014** | **1** | **1** | **Hypothetical protein MJ0597.** |
| **Q41046** | **1** | **1** | **Phytochrome.** |
| **Q10265** | **9** | **4** | **Probable heat shock protein C13G7.02C.** |
| **Q10127** | **1** | **1** | **Hypothetical zinc finger protein F56D1.1 in chromo** |
| **P35288** | **1** | **1** | **Ras-related protein Rab-23 (RAB-15).** |
| **O50290** | **6** | **2** | **ATP synthase beta chain (EC 3.6.3.14).** |
| **O81372;P47921;P47922;Q02254;O64903;P47923;Q01402** | **2** | **1** | **Nucleoside diphosphate kinase I (EC 2.7.4.6) (NDK** |
| **P23750** | **1** | **1** | **Histone H4.1.** |
| **O77788** | **3** | **3** | **Neurofilament triplet M protein (160 kDa neurofila** |
| **P50217** | **1** | **1** | **Isocitrate dehydrogenase [NADP] (EC 1.1.1.42) (Oxa** |
| **P19224** | **1** | **1** | **UDP-glucuronosyltransferase 1-6 precursor_ microso** |
| **P10577** | **1** | **1** | **Nitrogen assimilation regulatory protein.** |
| **O23755** | **9** | **5** | **Elongation factor 2 (EF-2).** |
| **Q01859;P37399;P00829;P56480;P00830;P22068;P49376;Q92G88;Q9TM41** | **8** | **1** | **ATP synthase beta chain_ mitochondrial precursor (** |
| **P05037** | **1** | **1** | **ATP synthase beta chain (EC 3.6.3.14).** |
| **P87197** | **2** | **1** | **Glyceraldehyde 3-phosphate dehydrogenase (EC 1.2.1** |
| **P40233** | **3** | **2** | **Casein kinase I homolog cki1 (EC 2.7.1.-).** |
| **Q9Z175** | **1** | **1** | **Lysyl oxidase homolog 3 precursor (EC 1.4.3.-) (Ly** |
| **Q58885** | **1** | **1** | **Dihydroorotase (EC 3.5.2.3) (DHOase).** |
| **O85282** | **8** | **3** | **Chaperone protein dnaK (Heat shock protein 70) (He** |
| **P26301;P25696;Q42971** | **7** | **2** | **Enolase 1 (EC 4.2.1.11) (2-phosphoglycerate dehydr** |
| **O68324** | **1** | **1** | **60 kDa chaperonin (Protein Cpn60) (groEL protein).** |
| **P15636** | **1** | **1** | **Protease I precursor (EC 3.4.21.50) (API) (Lysyl e** |
| **O60152** | **6** | **1** | **Probable N-end-recognizing protein (Ubiquitin-prot** |
| **P04445** | **1** | **1** | **Transcription factor 1.** |
| **P25858** | **7** | **1** | **Glyceraldehyde 3-phosphate dehydrogenase_ cytosoli** |
| **O34221** | **1** | **1** | **Flagellin C.** |
| **P40137** | **2** | **1** | **Adenylate cyclase 1 (EC 4.6.1.1) (ATP pyrophosphat** |
| **P26413** | **9** | **1** | **Heat shock 70 kDa protein.** |
| **P52410** | **1** | **1** | **3-oxoacyl-[acyl-carrier-protein] synthase I_ chlor** |
| **P23477** | **1** | **1** | **ATP-dependent nuclease subunit B.** |
| **P14776** | **1** | **1** | **Soluble hydrogenase_ small subunit (EC 1.12.-.-) (** |
| **Q56073;P04475** | **6** | **2** | **Chaperone protein dnaK (Heat shock protein 70) (He** |
| **O65735** | **2** | **1** | **Fructose-bisphosphate aldolase_ cytoplasmic isozym** |
| **Q9UKX2** | **3** | **1** | **Myosin heavy chain_ skeletal muscle_ adult 2 (Myos** |
| **Q9XCB1** | **7** | **3** | **Chaperone protein dnaK (Heat shock protein 70) (He** |
| **Q28288** | **1** | **1** | **Syntaxin binding protein 2 (Unc-18 homolog 2) (Unc** |
| **Q03684** | **6** | **2** | **Luminal binding protein 4 precursor (BiP 4) (78 kD** |
| **P11144** | **7** | **1** | **Heat shock 70 kDa protein (HSP70) (Cytoplasmic ant** |
| **P57593** | **2** | **2** | **Elongation factor G (EF-G).** |
| **Q58718** | **8** | **1** | **DNA double-strand break repair rad50 ATPase.** |
| **P41508** | **4** | **1** | **P115 protein.** |
| **P20363;O22349;P28752;P33627;Q02245;Q38771;Q43473;O22347;P05209;P08537;P14640;P14641;P29510;P29511;P33628;P33629;P36220;P46259;Q96460;Q9ZRB7;Q9ZRR5** | **3** | **2** | **Tubulin alpha-3/alpha-5 chain.** |
| **P36875** | **1** | **1** | **Protein phosphatase PP2A regulatory subunit A (PR6** |
| **P49467** | **2** | **1** | **DNA-directed RNA polymerase beta' chain (EC 2.7.7.** |
| **Q9T1V6** | **2** | **1** | **Protein gp42.** |
| **Q9RRX5** | **2** | **1** | **Phenylalanyl-tRNA synthetase beta chain (EC 6.1.1.** |
| **O49169** | **5** | **1** | **Elongation factor 1-alpha (EF-1-alpha).** |
| **P11857;P12460;P18025;P20365;P25862;P28551;P29501;P29515;P29516;P33630;P33631;P37392;P45960;P46265;P93176;Q04709;Q08115;Q41783;Q41784;Q43697;Q9ZPN7;Q9ZPN9;Q9ZPP0;Q9ZRA8;Q9ZRA9;Q9ZRB1;Q9ZRB2;Q9ZSW1** | **1** | **1** | **Tubulin beta chain (Beta-1 and beta-2).** |
| **P51554** | **5** | **1** | **Elongation factor 1-alpha (EF-1-alpha).** |
| **P41080** | **2** | **1** | **DNA gyrase subunit A (EC 5.99.1.3).** |
| **P11145** | **6** | **1** | **Heat shock 70 kDa protein 4 (HSP70).** |
| **Q9PB05** | **5** | **1** | **Chaperone protein dnaK (Heat shock protein 70) (He** |
| **Q09886** | **2** | **1** | **Hypothetical 13.7 kDa protein C584.12 in chromosom** |
| **P19450** | **5** | **3** | **Cellulose synthase operon C protein.** |
| **P06156;P12571;P14623** | **1** | **1** | **Fusion glycoprotein precursor [Contains: Fusion gl** |
| **P45482** | **1** | **1** | **Cell division protein ftsZ.** |
| **P04144;P04671** | **1** | **1** | **Nodulin 23 precursor (N-23).** |
| **Q9ZE22** | **1** | **1** | **50S ribosomal protein L10.** |
| **Q9HV43** | **3** | **1** | **Chaperone protein dnaK (Heat shock protein 70) (He** |
| **P29196** | **3** | **1** | **Phosphoenolpyruvate carboxylase (EC 4.1.1.31) (PEP** |
| **P70998** | **1** | **1** | **Spermidine synthase (EC 2.5.1.16) (Putrescine amin** |
| **P42794;P42795;P42796;P46287;O22540;Q10157** | **2** | **1** | **60S ribosomal protein L11 (L16).** |
| **O03064** | **1** | **1** | **ATP synthase beta chain (EC 3.6.3.14) (Fragment).** |
| **P08799** | **5** | **1** | **Myosin II heavy chain_ non muscle.** |
| **Q9TM34** | **2** | **1** | **DNA-directed RNA polymerase beta" chain (EC 2.7.7.** |
| **P13533;P02563;P13539;Q02566** | **3** | **1** | **Myosin heavy chain_ cardiac muscle alpha isoform (** |
| **Q00796** | **2** | **1** | **Sorbitol dehydrogenase (EC 1.1.1.14) (L-iditol 2-d** |
| **P32061** | **3** | **1** | **Acyl-[acyl-carrier protein] desaturase_ chloroplas** |
| **P50021** | **8** | **3** | **Chaperone protein dnaK2 (Heat shock protein 70-2)** |
| **P28919** | **3** | **3** | **Alkaline exonuclease (EC 3.1.11.-).** |
| **P06576** | **3** | **1** | **ATP synthase beta chain_ mitochondrial precursor (** |
| **P26519** | **4** | **1** | **Glyceraldehyde 3-phosphate dehydrogenase_ cytosoli** |
| **P37175** | **1** | **1** | **Protein ybaK.** |
| **O95198** | **2** | **1** | **Kelch-like protein 2 (Actin-binding protein Mayven** |
| **P08824** | **3** | **1** | **RuBisCO subunit binding-protein alpha subunit (60** |
| **P50019;P94317** | **3** | **1** | **Chaperone protein dnaK (Heat shock protein 70) (He** |
| **Q28247** | **3** | **1** | **Collagen alpha 5(IV) chain (Fragment).** |
| **Q9REQ3** | **2** | **1** | **DNA recombination protein rmuC homolog.** |
| **Q9ZHG4** | **2** | **1** | **DNA polymerase III alpha subunit (EC 2.7.7.7).** |
| **P25556;P25558** | **2** | **2** | **Hypothetical 25.8 kDa protein in BUD3-GBP2 interge** |
| **Q96551;Q96552** | **1** | **1** | **S-adenosylmethionine synthetase 1 (EC 2.5.1.6) (Me** |
| **P36399** | **1** | **1** | **Uracil phosphoribosyltransferase (EC 2.4.2.9) (UMP** |
| **P36604** | **4** | **1** | **78 kDa glucose-regulated protein homolog precursor** |
| **P23677** | **2** | **1** | **1D-myo-inositol-trisphosphate 3-kinase A (EC 2.7.1** |
| **O97125** | **8** | **2** | **Heat shock protein 68.** |
| **Q9JT81;Q9JY99** | **1** | **1** | **Chorismate synthase (EC 4.6.1.4) (5-enolpyruvylshi** |
| **Q02147** | **1** | **1** | **ATP phosphoribosyltransferase regulatory subunit.** |
| **P29309** | **2** | **1** | **14-3-3-like protein (Fragment).** |
| **P17820** | **2** | **1** | **Chaperone protein dnaK (Heat shock protein 70) (He** |
| **P02550;P02551;P02552;P04687;P05213;P05216** | **2** | **1** | **Tubulin alpha chain.** |
| **P02829;P15108** | **3** | **1** | **Heat shock protein HSP82.** |
| **P54774** | **1** | **1** | **Cell division cycle protein 48 homolog (Valosin co** |
| **P32062;Q43593** | **3** | **1** | **Acyl-[acyl-carrier protein] desaturase_ chloroplas** |
| **P48823** | **2** | **1** | **Beta-hexosaminidase A precursor (EC 3.2.1.52) (N-a** |
| **Q42662** | **1** | **1** | **5-methyltetrahydropteroyltriglutamate--homocystein** |
| **Q27727** | **1** | **1** | **Enolase (EC 4.2.1.11) (2-phosphoglycerate dehydrat** |
| **Q9UKX3** | **3** | **1** | **Myosin heavy chain_ skeletal muscle_ extraocular (** |
| **O72539** | **1** | **1** | **DNA polymerase (EC 2.7.7.7).** |
| **P22201;P23413;P92549;P05492;P05493;P05494;P15998;P24459;Q01915;Q06735** | **2** | **2** | **ATP synthase alpha chain_ mitochondrial (EC 3.6.3.** |
| **P29685** | **8** | **2** | **ATP synthase beta chain_ mitochondrial precursor (** |
| **P12882;Q28641** | **6** | **2** | **Myosin heavy chain_ skeletal muscle_ adult 1 (Myos** |
| **P46388** | **2** | **1** | **Chromosomal replication initiator protein dnaA.** |
| **O87384** | **2** | **1** | **Chaperone protein dnaK (Heat shock protein 70) (He** |
| **Q90593;P06761;Q91883** | **8** | **1** | **78 kDa glucose-regulated protein precursor (GRP 78** |
| **P07041** | **2** | **1** | **Histone H3.** |
| **P38482** | **7** | **2** | **ATP synthase beta chain_ mitochondrial precursor (** |
| **P57193** | **1** | **1** | **3-oxoacyl-[acyl-carrier-protein] synthase I (EC 2.** |
| **P04264** | **2** | **1** | **Keratin_ type II cytoskeletal 1 (Cytokeratin 1) (K** |
| **P08108** | **7** | **1** | **Heat shock cognate 70 kDa protein (HSP70).** |
| **P36067** | **1** | **1** | **Hypothetical 19.8 kDa protein in APL2-MYO3 interge** |
| **P82345** | **1** | **1** | **Hemoglobin beta-0 chain (HB 0).** |
| **P10992;P10993** | **1** | **1** | **Actin_ macronuclear.** |
| **P71331** | **7** | **1** | **Chaperone protein dnaK (Heat shock protein 70) (He** |
| **P42896;Q9LEI9;Q9LEJ0;Q43130;Q43321** | **6** | **1** | **Enolase (EC 4.2.1.11) (2-phosphoglycerate dehydrat** |
| **O10344** | **1** | **1** | **Late expression factor 5.** |
| **P25840** | **8** | **1** | **Heat shock 70 kDa protein.** |
| **P46212** | **1** | **1** | **Pyridoxal phosphate biosynthetic protein pdxJ (Fra** |
| **O22047** | **1** | **1** | **Chalcone synthase E (EC 2.3.1.74) (Naringenin-chal** |
| **P18615** | **1** | **1** | **RD protein.** |
| **P41887** | **2** | **1** | **Heat shock protein 90 homolog.** |
| **P52827;P52815;Q9DB15** | **2** | **1** | **60S ribosomal protein L12_ mitochondrial precursor** |
| **P95334** | **6** | **2** | **Chaperone protein dnaK (Heat shock protein 70) (He** |
| **P51538** | **2** | **1** | **Cytochrome P450 3A9 (EC 1.14.14.1) (CYPIIIA9) (P45** |
| **O50008** | **2** | **1** | **5-methyltetrahydropteroyltriglutamate--homocystein** |
| **P11147** | **5** | **2** | **Heat shock 70 kDa protein cognate 4 (Heat shock 70** |
| **P10112** | **1** | **1** | **Beta crystallin S (Gamma crystallin S).** |
| **P27541** | **6** | **1** | **Heat shock 70 kDa protein.** |
| **P19208** | **7** | **1** | **Heat shock 70 kDa protein C precursor (Fragment).** |
| **Q04647** | **3** | **1** | **Probable cytochrome c biosynthesis protein.** |
| **P25379** | **2** | **1** | **Catabolic L-serine/threonine dehydratase [Includes** |
| **P03993;P08565;P14624;P15174;P23324;P42739;P49634;P02248;P04838;P08618;P13117;P14792;P19848;P20685;P22589;P23398;P42740;P46574;P49635;Q05550;Q9VZL4** | **3** | **1** | **Ubiquitin.** |
| **P46493** | **1** | **1** | **Threonine dehydratase biosynthetic (EC 4.2.1.16) (** |
| **P24067;O24581** | **9** | **2** | **Luminal binding protein 2 precursor (BiP2) (Heat s** |
| **Q27139** | **2** | **1** | **Elongation factor 1-alpha 1 (EF-1-alpha-1).** |
| **P22623** | **6** | **1** | **Heat shock 70 kDa protein II (HSP70 II) (Fragment)** |
| **O66680** | **1** | **1** | **Leucyl-tRNA synthetase alpha subunit (EC 6.1.1.4)** |
| **P08927** | **10** | **2** | **RuBisCO subunit binding-protein beta subunit_ chlo** |
| **Q91233** | **4** | **1** | **Heat shock 70 kDa protein (HSP70).** |
| **P05456** | **7** | **1** | **Heat shock 70 kDa protein.** |
| **P46815** | **2** | **1** | **Antigen 84.** |
| **P54652;P17156;P34933;P14659** | **12** | **2** | **Heat shock-related 70 kDa protein 2 (Heat shock 70** |
| **P50346** | **3** | **1** | **60S acidic ribosomal protein P0.** |
| **O00445** | **1** | **0** | **Synaptotagmin V (SytV).** |
| **O02192** | **2** | **0** | **Heat shock protein 83 (HSP 82).** |
| **O02608** | **1** | **0** | **Phosphoglycerate kinase (EC 2.7.2.3).** |
| **O06594** | **1** | **0** | **Nicotinate-nucleotide pyrophosphorylase [carboxyla** |
| **O13710** | **1** | **0** | **DNA repair protein spr18 (SMC partner of rad18).** |
| **O22582;P40283;P93354** | **2** | **0** | **Histone H2B.** |
| **O24047;Q08062;O48905** | **8** | **0** | **Malate dehydrogenase_ cytoplasmic (EC 1.1.1.37).** |
| **O24415** | **1** | **0** | **60S acidic ribosomal protein P2B.** |
| **O32264** | **1** | **0** | **Probable 2-ketogluconate reductase (EC 1.1.1.215)** |
| **O32464** | **4** | **0** | **Chaperone protein dnaK (Heat shock protein 70) (He** |
| **O33099** | **1** | **0** | **Probable tRNA (5-methylaminomethyl-2-thiouridylate** |
| **O43464** | **1** | **0** | **Serine protease HTRA2 (EC 3.4.21.-) (Omi stress-re** |
| **O43603** | **1** | **0** | **Galanin receptor type 2 (GAL2-R) (GALR2).** |
| **O49998** | **2** | **0** | **14-3-3-like protein F.** |
| **O50487;P41054;P48294;P48295;P95846** | **1** | **0** | **RecA protein (Recombinase A).** |
| **O54699** | **1** | **0** | **Equilibrative nucleoside transporter 2 (Equilibrat** |
| **O64937** | **6** | **0** | **Elongation factor 1-alpha (EF-1-alpha).** |
| **O65195** | **1** | **0** | **Myo-inositol-1-phosphate synthase (EC 5.5.1.4) (MI** |
| **O67077** | **1** | **0** | **Cell division protein ftsH homolog (EC 3.4.24.-).** |
| **O67137** | **1** | **0** | **DNA gyrase subunit B (EC 5.99.1.3).** |
| **O67809** | **1** | **0** | **30S ribosomal protein S2.** |
| **O75891** | **1** | **0** | **10-formyltetrahydrofolate dehydrogenase (EC 1.5.1.** |
| **O83084** | **1** | **0** | **Glucose inhibited division protein A.** |
| **O95753** | **1** | **0** | **TATA box binding protein-like protein 1 (TBP-like** |
| **P00360** | **2** | **0** | **Glyceraldehyde 3-phosphate dehydrogenase 1 (EC 1.2** |
| **P00883;P04075;P05064;P05065** | **1** | **0** | **Fructose-bisphosphate aldolase A (EC 4.1.2.13) (Mu** |
| **P04127** | **1** | **0** | **PAP fimbrial major pilin protein precursor (PAP pi** |
| **P04422;P33109** | **1** | **0** | **Aspartate ammonia-lyase (EC 4.3.1.1) (Aspartase).** |
| **P04765;P10630;P29562;Q14240** | **2** | **0** | **Eukaryotic initiation factor 4A-I (eIF-4A-I) (eIF4** |
| **P05184** | **1** | **0** | **Cytochrome P450 3A3 (EC 1.14.14.1) (CYPIIIA3) (HLp** |
| **P05440** | **1** | **0** | **ATP synthase beta chain (EC 3.6.3.14).** |
| **P05833** | **2** | **0** | **RepA protein.** |
| **P05981** | **2** | **0** | **Serine protease hepsin (EC 3.4.21.-) (Transmembran** |
| **P06148** | **1** | **0** | **Hemoglobin F-I.** |
| **P06805** | **4** | **0** | **Elongation factor 1-alpha (EF-1-alpha).** |
| **P06826;P07381;P15660;P15661;P15663;P15664;P15665;P15666;P15667;P15668;P15669;P15670;P15675;P15676;P15677;P15678;P15680;P15683;P16978;P16984;P16985;P16988;P18277;P26053;P26054;P26059;P26061;P26062;P26063;P26064;P26065;P26066;P26077;P26078;P26079;P26080;P26081** | **1** | **0** | **Nucleoprotein.** |
| **P06977;P24165;P24746** | **2** | **0** | **Glyceraldehyde 3-phosphate dehydrogenase A (EC 1.2** |
| **P07345** | **1** | **0** | **Tryptophan synthase beta chain (EC 4.2.1.20).** |
| **P07823;P11021;P20029** | **7** | **0** | **78 kDa glucose-regulated protein precursor (GRP 78** |
| **P08109;P11142;P19120;P19378** | **7** | **0** | **Heat shock cognate 71 kDa protein.** |
| **P08490** | **1** | **0** | **Genome polyprotein [Contains: Genome-linked protei** |
| **P08593** | **1** | **0** | **MSS18 protein.** |
| **P08800** | **1** | **0** | **UTP--glucose-1-phosphate uridylyltransferase (EC 2** |
| **P08862** | **1** | **0** | **Excisase A (NifD element site-specific recombinase** |
| **P09189;O65719** | **15** | **0** | **Heat shock cognate 70 kDa protein.** |
| **P09446** | **6** | **0** | **Heat shock 70 kDa protein A.** |
| **P09746** | **1** | **0** | **Shufflon protein A'.** |
| **P10675** | **1** | **0** | **Fasciclin I precursor (FAS I) (FCN).** |
| **P10719** | **3** | **0** | **ATP synthase beta chain_ mitochondrial precursor (** |
| **P11055** | **1** | **0** | **Myosin heavy chain_ fast skeletal muscle_ embryoni** |
| **P11141** | **1** | **0** | **Heat shock 70 kDa protein F_ mitochondrial precurs** |
| **P11464** | **1** | **0** | **Pregnancy-specific beta-1-glycoprotein 1 precursor** |
| **P11559** | **1** | **0** | **Methyl-coenzyme M reductase alpha subunit (EC 1.8.** |
| **P12783** | **1** | **0** | **Phosphoglycerate kinase_ cytosolic (EC 2.7.2.3).** |
| **P13290** | **1** | **0** | **Glycoprotein G.** |
| **P13547** | **1** | **0** | **ATP synthase protein 9_ mitochondrial (EC 3.6.3.14** |
| **P15254** | **3** | **0** | **Phosphoribosylformylglycinamidine synthase (EC 6.3** |
| **P15630** | **1** | **0** | **Hypothetical protein 2 (ORF II).** |
| **P16627;P55063** | **7** | **0** | **Heat shock-related 70 kDa protein.** |
| **P16989** | **1** | **0** | **DNA-binding protein A (Cold shock domain protein A** |
| **P17066** | **9** | **0** | **Heat shock 70 kDa protein 6 (Heat shock 70 kDa pro** |
| **P17507** | **5** | **0** | **Elongation factor 1-alpha_ oocyte form (EF-1-alpha** |
| **P17614** | **6** | **0** | **ATP synthase beta chain_ mitochondrial precursor (** |
| **P17879;Q07439** | **7** | **0** | **Heat shock 70 kDa protein 1 (HSP70.1) (HSP70-1/HSP** |
| **P18623** | **1** | **0** | **Viomycin phosphotransferase (EC 2.7.1.103) (Viomyc** |
| **P18624** | **5** | **0** | **Elongation factor 1-alpha (EF-1-alpha) (50 kDa act** |
| **P19023** | **6** | **0** | **ATP synthase beta chain_ mitochondrial precursor (** |
| **P19089** | **1** | **0** | **Glyceraldehyde 3-phosphate dehydrogenase (EC 1.2.1** |
| **P19177;P25470;P40280;P40281** | **1** | **0** | **Histone H2A.** |
| **P19415** | **1** | **0** | **Pesticidial crystal protein cry1Da (Insecticidal d** |
| **P19690** | **1** | **0** | **RecA protein (Recombinase A).** |
| **P19993;Q00488** | **2** | **0** | **Chaperone protein dnaK (Heat shock protein 70) (He** |
| **P20442** | **3** | **0** | **Chaperone protein dnaK (Heat shock protein 70) (He** |
| **P21302;Q01325;Q01327;Q9JTI4;Q9JYI3** | **1** | **0** | **Ornithine carbamoyltransferase_ catabolic (EC 2.1.** |
| **P22132;Q96481** | **1** | **0** | **Actin 2.** |
| **P22258** | **1** | **0** | **Cell surface protein precursor (S-layer protein).** |
| **P22274** | **1** | **0** | **ADP-ribosylation factor.** |
| **P22337** | **3** | **0** | **Acyl-[acyl-carrier protein] desaturase_ chloroplas** |
| **P22349** | **1** | **0** | **Hypothetical 46.2 kDa protein in purE 3'region (OR** |
| **P22774** | **3** | **0** | **Heat shock 70 kDa protein_ mitochondrial precursor** |
| **P22882** | **1** | **0** | **17 kDa surface antigen precursor.** |
| **P22953** | **12** | **0** | **Heat shock cognate 70 kDa protein 1 (Hsc70.1).** |
| **P23148** | **1** | **0** | **Acyl transferase (EC 2.3.1.-) (ACT) (Myristoyl-ACP** |
| **P23343;** | **5** | **0** | **Actin 1.** |
| **P23669** | **1** | **0** | **Threonine synthase (EC 4.2.99.2).** |
| **P24481** | **1** | **0** | **Phenylalanine ammonia-lyase 1 (EC 4.3.1.5).** |
| **P24517;P24554** | **1** | **0** | **DNA repair protein radA (DNA repair protein sms).** |
| **P24525** | **1** | **0** | **Peptidyl-prolyl cis-trans isomerase (EC 5.2.1.8) (** |
| **P24543** | **1** | **0** | **RecA protein (Recombinase A).** |
| **P24706** | **1** | **0** | **Superoxide dismutase [Cu-Zn] (EC 1.15.1.1).** |
| **P25166** | **5** | **0** | **Elongation factor 1-alpha (EF-1-alpha).** |
| **P25698;P29521** | **6** | **0** | **Elongation factor 1-alpha (EF-1-alpha).** |
| **P25719;P22011** | **2** | **0** | **Peptidyl-prolyl cis-trans isomerase C_ mitochondri** |
| **P26300** | **5** | **0** | **Enolase (EC 4.2.1.11) (2-phosphoglycerate dehydrat** |
| **P26791** | **3** | **0** | **Heat shock 70 kDa protein.** |
| **P27322** | **10** | **0** | **Heat shock cognate 70 kDa protein 2.** |
| **P27420** | **7** | **0** | **Heat shock 70 kDa protein C precursor.** |
| **P27583** | **1** | **0** | **3-oxoacyl-[acyl-carrier protein] reductase (EC 1.1** |
| **P28241** | **1** | **0** | **Isocitrate dehydrogenase [NAD] subunit 2_ mitochon** |
| **P29108;P22243;P28645;Q41319;Q96456** | **3** | **0** | **Acyl-[acyl-carrier protein] desaturase_ chloroplas** |
| **P29251** | **2** | **0** | **Folic acid synthesis protein [Includes: Dihydroneo** |
| **P29357** | **10** | **0** | **Chloroplast envelope membrane 70 kDa heat shock-re** |
| **P29473** | **1** | **0** | **Nitric-oxide synthase_ endothelial (EC 1.14.13.39)** |
| **P29710** | **1** | **0** | **ATP synthase gamma chain_ sodium ion specific (EC** |
| **P29766;P46286** | **1** | **0** | **60S ribosomal protein L2 (L8) (Ribosomal protein T** |
| **P30722** | **2** | **0** | **Chaperone protein dnaK (Heat shock protein 70) (He** |
| **P30755;O49118;P05621;P27807;P30756;P49120;P54348;Q43261** | **2** | **0** | **Histone H2B.1.** |
| **P31018** | **2** | **0** | **Elongation factor 1-alpha (EF-1-alpha).** |
| **P31543** | **1** | **0** | **Heat shock protein 100 (CLP protein).** |
| **P31638** | **1** | **0** | **Acetyl-coenzyme A synthetase (EC 6.2.1.1) (Acetate** |
| **P33207** | **1** | **0** | **3-oxoacyl-[acyl-carrier protein] reductase_ chloro** |
| **P33887** | **1** | **0** | **Eukaryotic translation initiation factor 2 subunit** |
| **P34385** | **1** | **0** | **Hypothetical 66.5 kDa protein F02A9.4A in chromoso** |
| **P34794** | **6** | **0** | **RuBisCO subunit binding-protein alpha subunit_ chl** |
| **P34817** | **1** | **0** | **Tryptophan synthase beta chain (EC 4.2.1.20).** |
| **P34824** | **7** | **0** | **Elongation factor 1-alpha (EF-1-alpha).** |
| **P34930** | **8** | **0** | **Heat shock 70 kDa protein 1 (HSP70.1).** |
| **P35016** | **5** | **0** | **Endoplasmin homolog precursor (GRP94 homolog).** |
| **P35017** | **2** | **0** | **Superoxide dismutase [Mn]_ mitochondrial precursor** |
| **P35844** | **1** | **0** | **KES1 protein.** |
| **P36183** | **4** | **0** | **Endoplasmin homolog precursor (GRP94 homolog).** |
| **P36415** | **6** | **0** | **Heat shock cognate protein (Aginactin).** |
| **P37899** | **3** | **0** | **Heat shock 70 kDa protein.** |
| **P41166** | **3** | **0** | **Elongation factor 1-alpha (EF-1-alpha).** |
| **P41797** | **7** | **0** | **Heat shock protein SSA1.** |
| **P42340** | **1** | **0** | **Chloroplast 50S ribosomal protein L14 (Fragments).** |
| **P42374** | **3** | **0** | **Chaperone protein dnaK (Heat shock protein 70) (He** |
| **P42500** | **1** | **0** | **Phytochrome A.** |
| **P42643** | **3** | **0** | **14-3-3-like protein GF14 chi (General regulatory f** |
| **P43643** | **6** | **0** | **Elongation factor 1-alpha (EF-1-alpha) (Vitronecti** |
| **P43700** | **1** | **0** | **DNA gyrase subunit A (EC 5.99.1.3).** |
| **P43910** | **1** | **0** | **L-seryl-tRNA(Sec) selenium transferase (EC 2.9.1.1** |
| **P44669** | **2** | **0** | **Chaperone protein hscA homolog.** |
| **P44781** | **2** | **0** | **RNA polymerase associated protein homolog (ATP-dep** |
| **P44785** | **1** | **0** | **ATP-binding protein abc.** |
| **P45323** | **1** | **0** | **Molybdate-binding periplasmic protein precursor.** |
| **P45554** | **2** | **0** | **Chaperone protein dnaK (Heat shock protein 70) (He** |
| **P45958** | **4** | **0** | **Chaperone protein dnaK (Heat shock protein 70) (He** |
| **P46226** | **1** | **0** | **Triosephosphate isomerase_ cytosolic (EC 5.3.1.1)** |
| **P46288** | **1** | **0** | **60S ribosomal protein L27.** |
| **P46563;P17784;P46256** | **3** | **0** | **Fructose-bisphosphate aldolase 2 (EC 4.1.2.13) (Al** |
| **P46598** | **3** | **0** | **Heat shock protein 90 homolog.** |
| **P46600** | **1** | **0** | **Homeobox-leucine zipper protein HAT1 (HD-ZIP prote** |
| **P46683** | **1** | **0** | **Ankyrin repeat-containing protein YAR1.** |
| **P46835** | **1** | **0** | **DNA polymerase I (EC 2.7.7.7) (POL I).** |
| **P46850** | **1** | **0** | **Protein rtcB.** |
| **P47394** | **1** | **0** | **Hypothetical protein MG148.** |
| **P47766** | **1** | **0** | **DNA-directed RNA polymerase beta chain (EC 2.7.7.6** |
| **P47771** | **1** | **0** | **Aldehyde dehydrogenase [NAD(P)+] 1 (EC 1.2.1.5).** |
| **P47773** | **6** | **0** | **Heat shock cognate 71 kDa protein.** |
| **P48347** | **2** | **0** | **14-3-3-like protein GF14 epsilon (General regulato** |
| **P48494;P48495** | **2** | **0** | **Triosephosphate isomerase_ cytosolic (EC 5.3.1.1)** |
| **P48720** | **7** | **0** | **Heat shock 70 kDa protein.** |
| **P49118** | **5** | **0** | **Luminal binding protein precursor (BiP) (78 kDa gl** |
| **P49770** | **1** | **0** | **Translation initiation factor eIF-2B beta subunit** |
| **P50867** | **1** | **0** | **Cysteine synthase (EC 4.2.99.8) (O-acetylserine su** |
| **P50917** | **1** | **0** | **Malate dehydrogenase (EC 1.1.1.37).** |
| **P51913** | **1** | **0** | **Alpha enolase (EC 4.2.1.11) (2-phospho-D-glycerate** |
| **P53421** | **5** | **0** | **Heat-shock protein 70 1 (HSP72).** |
| **P53456;P14227** | **2** | **0** | **Actin 2.** |
| **P53623** | **7** | **0** | **Heat shock protein 70 2.** |
| **P53691** | **1** | **0** | **Peptidyl-prolyl cis-trans isomerase CPR6 (EC 5.2.1** |
| **P54221** | **1** | **0** | **Seryl-tRNA synthetase (EC 6.1.1.11) (Serine--tRNA** |
| **P54651** | **2** | **0** | **Heat shock cognate 90 kDa protein.** |
| **P54793** | **1** | **0** | **Arylsulfatase F precursor (EC 3.1.6.-) (ASF).** |
| **P54936** | **1** | **0** | **LIN-2 protein.** |
| **P55361** | **1** | **0** | **Probable transcriptional regulator syrB.** |
| **P55737** | **4** | **0** | **Heat shock protein 81-2 (HSP81-2).** |
| **P55964** | **1** | **0** | **Pyruvate kinase isozyme G_ chloroplast (EC 2.7.1.4** |
| **P56133;Q9ZKC2** | **1** | **0** | **Protein-L-isoaspartate O-methyltransferase (EC 2.1** |
| **P57098** | **2** | **0** | **Glycerate kinase (EC 2.7.1.31).** |
| **P57491** | **1** | **0** | **CTP synthase (EC 6.3.4.2) (UTP--ammonia ligase) (C** |
| **P57870** | **4** | **0** | **Chaperone protein dnaK (Heat shock protein 70) (He** |
| **P58593** | **2** | **0** | **EPS I polysaccharide export protein epsB.** |
| **P71585** | **1** | **0** | **Probable serine/threonine-protein kinase pknA (EC** |
| **P72580** | **2** | **0** | **Prenyl transferase (EC 2.5.1.-).** |
| **P72872** | **1** | **0** | **Hypothetical 37.9 kDa protein SLL0926.** |
| **P74391** | **1** | **0** | **DNA repair protein radA homolog (DNA repair protei** |
| **P75344** | **2** | **0** | **Chaperone protein dnaK (Heat shock protein 70) (He** |
| **P77510** | **1** | **0** | **Sensor kinase dpiB (EC 2.7.3.-) (Sensor kinase cit** |
| **P79920** | **1** | **0** | **Atonal protein homolog 3 (Helix-loop-helix protein** |
| **P80483** | **1** | **0** | **Peridinin-chlorophyll A binding protein 3 (PCP).** |
| **P81042** | **1** | **0** | **Hemoglobin epsilon chain (Fragment).** |
| **P82556** | **1** | **0** | **Ribosome recycling factor (Ribosome releasing fact** |
| **P87553** | **1** | **0** | **DNA polymerase (EC 2.7.7.7).** |
| **P91902** | **5** | **0** | **Heat shock protein 70 (HSP70).** |
| **P93819** | **6** | **0** | **Malate dehydrogenase_ cytoplasmic 1 (EC 1.1.1.37).** |
| **P97043** | **2** | **0** | **Probable tRNA modification GTPase trmE.** |
| **Q9CK66** | **1** | **0** | **L-seryl-tRNA(Sec) selenium transferase (EC 2.9.1.1** |
| **Q9K6Y0** | **2** | **0** | **Excinuclease ABC subunit A.** |
| **Q9KE51** | **3** | **0** | **Chaperone protein htpG (Heat shock protein htpG) (** |
| **Q9N1Q9** | **1** | **0** | **Calcium-binding protein CaBP2.** |
| **Q9PHZ3** | **2** | **0** | **Chaperone protein htpG (Heat shock protein htpG) (** |
| **Q9PKB9** | **1** | **0** | **Chromosomal replication initiator protein dnaA2.** |
| **Q9PQF2** | **4** | **0** | **Chaperone protein dnaK (Heat shock protein 70) (He** |
| **Q9RWH0** | **1** | **0** | **V-type ATP synthase subunit C (EC 3.6.3.14) (V-typ** |
| **Q9SML8;P57106** | **8** | **0** | **Malate dehydrogenase_ cytoplasmic (EC 1.1.1.37).** |
| **Q9SZW5** | **1** | **0** | **Potential cadmium/zinc-transporting ATPase 4 (EC 3** |
| **Q9ULD4** | **2** | **0** | **Bromodomain and PHD finger-containing protein 3 (F** |
| **Q9VNA8** | **1** | **0** | **Downstream of son gene protein homolog.** |
| **Q9VW43** | **1** | **0** | **Probable cytochrome P450 305a1 (EC 1.14.-.-) (CYPC** |
| **Q9VWA2** | **1** | **0** | **Probable deoxycytidylate deaminase (EC 3.5.4.12) (** |
| **Q9X1Y4** | **1** | **0** | **Elongation factor G like protein.** |
| **Q9X4T4** | **1** | **0** | **Phosphate transport system protein phoU.** |
| **Q9XDH6** | **2** | **0** | **DNA polymerase III alpha subunit (EC 2.7.7.7).** |
| **Q9Y623** | **3** | **0** | **Myosin heavy chain_ skeletal muscle_ fetal (Myosin** |
| **Q9ZK62** | **1** | **0** | **Signal recognition particle protein (Fifty-four ho** |
| **Q9ZLM1** | **1** | **0** | **DNA ligase (EC 6.5.1.2) (Polydeoxyribonucleotide s** |
| **Q00043** | **4** | **0** | **Heat shock 70 kDa protein.** |
| **Q93MD5** | **1** | **0** | **Hypothetical protein PCP12.** |
| **Q99KF0** | **2** | **0** | **Caspase recruitment domain protein 14 (Bcl10-inter** |
| **Q01077;P00358;P00359;P17819** | **3** | **0** | **Glyceraldehyde 3-phosphate dehydrogenase 2 (EC 1.2** |
| **Q01390** | **1** | **0** | **Sucrose synthase (EC 2.4.1.13) (Sucrose-UDP glucos** |
| **Q01877;P34931** | **9** | **0** | **Heat shock protein HSS1.** |
| **Q02748** | **2** | **0** | **Eukaryotic initiation factor 4A (eIF-4A) (eIF4A).** |
| **Q02779** | **1** | **0** | **Mitogen-activated protein kinase kinase kinase 10** |
| **Q03262** | **1** | **0** | **Hypothetical 71.1 kDa protein in DSK2-CAT8 interge** |
| **Q03606** | **1** | **0** | **Hypothetical 84.0 kDa protein T23G5.2 in chromosom** |
| **Q05825;Q24751** | **3** | **0** | **ATP synthase beta chain_ mitochondrial precursor (** |
| **Q10419** | **2** | **0** | **Mesentericin Y105 secretion protein mesE.** |
| **Q11090** | **1** | **0** | **Putative serine/threonine-protein kinase C01C4.3 i** |
| **Q14774** | **2** | **0** | **Homeobox protein HLX1 (Homeobox protein HB24).** |
| **Q19706** | **1** | **0** | **Probable eukaryotic translation initiation factor** |
| **Q25117** | **3** | **0** | **ATP synthase beta chain_ mitochondrial precursor (** |
| **Q26540** | **2** | **0** | **14-3-3 protein homolog 1.** |
| **Q27140** | **1** | **0** | **Elongation factor 1-alpha 2 (EF-1-alpha-2).** |
| **Q27975;P08107;Q27965;Q28222** | **7** | **0** | **Heat shock 70 kDa protein 1 (HSP70-1).** |
| **Q29465** | **1** | **0** | **Tyrosyl-tRNA synthetase (EC 6.1.1.1) (Tyrosyl--tRN** |
| **Q40034;P17786;Q03033;Q41803;P13905** | **7** | **0** | **Elongation factor 1-alpha (EF-1-alpha).** |
| **Q40608** | **1** | **0** | **ATP synthase B' chain (EC 3.6.3.14) (Subunit II).** |
| **Q42434** | **8** | **0** | **Luminal binding protein precursor (BiP) (78 kDa gl** |
| **Q43831** | **2** | **0** | **RuBisCO subunit binding-protein beta subunit (60 k** |
| **Q45551;Q9KWS7** | **6** | **0** | **Chaperone protein dnaK (Heat shock protein 70) (He** |
| **Q46438** | **2** | **0** | **Virulence plasmid protein pGP2-D.** |
| **Q50729** | **1** | **0** | **GMP synthase [glutamine-hydrolyzing] (EC 6.3.5.2)** |
| **Q52377** | **1** | **0** | **Excinuclease ABC subunit C (Fragment).** |
| **Q53200** | **1** | **0** | **Putative insertion sequence ATP-binding protein Y4** |
| **Q57633** | **1** | **0** | **Hypothetical ATP-binding protein MJ0169.** |
| **Q57670** | **1** | **0** | **V-type ATP synthase alpha chain (EC 3.6.3.14) (V-t** |
| **Q57907** | **1** | **0** | **Hypothetical protein MJ0483.** |
| **Q58283** | **2** | **0** | **Hypothetical ABC transporter ATP-binding protein M** |
| **Q58524** | **1** | **0** | **Putative ski2-type helicase (EC 3.6.1.-) [Contains** |
| **Q59906** | **1** | **0** | **Glyceraldehyde 3-phosphate dehydrogenase (EC 1.2.1** |
| **Q90339** | **1** | **0** | **Myosin heavy chain_ fast skeletal muscle.** |
| **Q90473** | **4** | **0** | **Heat shock cognate 71 kDa protein.** |
| **Q91291** | **3** | **0** | **Heat shock 70 kDa protein (HSP70).** |
| **Q91896** | **2** | **0** | **14-3-3 protein zeta.** |
| **Q92005** | **5** | **0** | **Elongation factor 1-alpha (EF-1-alpha).** |
| **Q92263** | **1** | **0** | **Glyceraldehyde 3-phosphate dehydrogenase (EC 1.2.1** |
| **Q96482** | **2** | **0** | **Actin 41 (Fragment).** |
| **Q96528** | **3** | **0** | **Catalase 1 (EC 1.11.1.6).** |
| **Q99002** | **2** | **0** | **14-3-3 protein homolog (TH1433).** |
| **Q99801** | **1** | **0** | **Homeobox protein NKX-3.1.** |
